# RNAProt: an efficient and feature-rich RNA binding protein binding site predictor

**DOI:** 10.1093/gigascience/giab054

**Published:** 2021-08-18

**Authors:** Michael Uhl, Van Dinh Tran, Florian Heyl, Rolf Backofen

**Affiliations:** Bioinformatics Group, Department of Computer Science, University of Freiburg, Georges-Koehler-Allee 106, 79110 Freiburg, Germany; Bioinformatics Group, Department of Computer Science, University of Freiburg, Georges-Koehler-Allee 106, 79110 Freiburg, Germany; Bioinformatics Group, Department of Computer Science, University of Freiburg, Georges-Koehler-Allee 106, 79110 Freiburg, Germany; Bioinformatics Group, Department of Computer Science, University of Freiburg, Georges-Koehler-Allee 106, 79110 Freiburg, Germany; Signalling Research Centres BIOSS and CIBSS, University of Freiburg, Schaenzlestr. 18, 79104 Freiburg, Germany

**Keywords:** CLIP-seq, eCLIP, RBP binding site prediction, deep learning, recurrent neural networks, visualization

## Abstract

**Background:**

Cross-linking and immunoprecipitation followed by next-generation sequencing (CLIP-seq) is the state-of-the-art technique used to experimentally determine transcriptome-wide binding sites of RNA-binding proteins (RBPs). However, it relies on gene expression, which can be highly variable between conditions and thus cannot provide a complete picture of the RBP binding landscape. This creates a demand for computational methods to predict missing binding sites. Although there exist various methods using traditional machine learning and lately also deep learning, we encountered several problems: many of these are not well documented or maintained, making them difficult to install and use, or are not even available. In addition, there can be efficiency issues, as well as little flexibility regarding options or supported features.

**Results:**

Here, we present RNAProt, an efficient and feature-rich computational RBP binding site prediction framework based on recurrent neural networks. We compare RNAProt with 1 traditional machine learning approach and 2 deep-learning methods, demonstrating its state-of-the-art predictive performance and better run time efficiency. We further show that its implemented visualizations capture known binding preferences and thus can help to understand what is learned. Since RNAProt supports various additional features (including user-defined features, which no other tool offers), we also present their influence on benchmark set performance. Finally, we show the benefits of incorporating additional features, specifically structure information, when learning the binding sites of an hairpin loop binding RBP.

**Conclusions:**

RNAProt provides a complete framework for RBP binding site predictions, from data set generation over model training to the evaluation of binding preferences and prediction. It offers state-of-the-art predictive performance, as well as superior run time efficiency, while at the same time supporting more features and input types than any other tool available so far. RNAProt is easy to install and use, comes with comprehensive documentation, and is accompanied by informative statistics and visualizations. All this makes RNAProt a valuable tool to apply in future RBP binding site research.

## Introduction

RNA-binding proteins (RBPs) regulate many vital steps in the RNA life cycle, such as splicing, transport, stability, and translation [1]. Recent studies suggest there are more than 2,000 human RBPs, including hundreds of unconventional RBPs, such as those lacking known RNA-binding domains [2–4]. Numerous RBPs have been implicated in diseases like cancer, neurodegeneration, and genetic disorders [5–7], lending urgency characterizing their functions and shedding light on their complex cellular interplay.

An important step to understanding RBP functions is to identify the precise RBP binding locations on regulated RNAs. In this regard, CLIP-seq (cross-linking and immunoprecipitation followed by next-generation sequencing) [8], together with its popular modifications photoactivatable-ribonucleoside-enhanced cross-linking and immunoprecipitation (PAR-CLIP) [9], individual-nucleotide resolution UV cross-linking and immunoprecipitation (iCLIP) [10], and enhanced CLIP (eCLIP) [11], has become the state-of-the-art technique used to experimentally determine transcriptome-wide binding sites of RBPs. A CLIP-seq experiment for a specific RBP results in a library of reads bound and protected by the RBP, making it possible to deduce its binding sites by mapping the reads back to the respective reference genome or transcriptome. In practice, a computational analysis of CLIP-seq data has to be adapted for each CLIP-seq protocol [12]. Within the analysis, arguably the most critical part is the process of peak calling: that is, inferring RBP binding sites from the mapped read profiles. Among the many existing peak callers, some popular tools are Piranha [13], CLIPper [14], and PureCLIP [15].

While peak calling is essential to separate authentic binding sites from unspecific interactions and thus reduce the false positive rate, it cannot solve the problem of expression dependency. In order to detect RBP binding sites by CLIP-seq, the target RNA has to be expressed at a certain level in the experiment. Since gene expression naturally varies between conditions, CLIP-seq data cannot be used directly to make condition-independent binding assumptions on a transcriptome-wide scale. Doing so would only increase the false negative rate: for example, marking all regions not covered by CLIP-seq reads as non-binding, while in fact one cannot tell due to the lack of expression information. Moreover, expression variation is especially high for long non-coding RNAs, an abundant class of non-coding RNAs gaining more and more attention due to their diverse cellular roles [16]. It is therefore of great importance to infer RBP binding characteristics from CLIP-seq data in order to predict missing binding sites. To give an example, Ferrarese et al. [17] investigated the role of the splicing factor Polypyrimidine tract-binding protein 1 (PTBP1) in differential splicing of the tumor suppressor gene Annexin A1 (ANXA7) in glioblastoma. Despite strong biological evidence for PTBP1 directly binding ANXA7, no binding site was found in a publicly available CLIP-seq data set for PTBP1. Instead, only a computational analysis was capable to detect and correctly localize the presence of potential binding sites, which were then experimentally validated.

Over the years, many approaches for RBP binding site prediction have been presented, from simple sequence motif searches to more sophisticated methods incorporating classical machine learning and, lately, also deep learning. Some popular earlier methods include RNAcontext [18] and GraphProt [19], which can both incorporate RNA sequence and structure information into their predictive models. While RNAcontext utilizes a sequence and structure motif model, GraphProt uses a graph kernel coupled with Support Vector Machine, showing improved performance over motif-based techniques. From 2015 on, various deep learning–based methods have been proposed, starting with DeepBind [20], which uses sequence information to train a convolutional neural network (CNN). Subsequent methods largely built upon this methodology, often using CNNs in combination with recurrent neural networks (RNNs) [21]. Some of them also incorporate additional features, usually specializing in a specific feature, such as structure, evolutionary conservation, or region type information, to demonstrate its benefits. While these methods can certainly provide state-of-the-art predictive performance, we encountered several issues: many lack proper documentation, are not maintained, or are not even available, even though they are presented as prediction tools in the original papers. Moreover, efficiency in terms of run time can be a problem, as well as restricted options regarding data processing and, in general, only a few supported features.

Here, we present RNAProt, a computational RBP binding site prediction framework based on RNNs that takes care of the described issues: RNAProt provides both state-of-the-art performance and efficient run times. It comes with comprehensive documentation and is easy to install via Conda. The availability of a Conda package, which no other related deep-learning tool offers to our knowledge, also allows for easy integration into larger workflows, such as Snakemake pipelines [22] or inside the Galaxy framework [23]. RNAProt offers various position-wise features on top of the sequence information, such as secondary structure, conservation scores, or region annotations, which can also be user supplied. Through its use of an RNN-based architecture, RNAProt natively supports input sequences of variable lengths. In contrast, CNNs are constrained to fixed-sized inputs that, for example, exclude the direct usage of variable-sized inputs, usually defined by peak callers. Moreover, RNAProt is currently the most flexible method with regard to the support of input data types: apart from sequences and genomic regions, it can also handle transcript regions, providing automatic feature annotations for all 3 types. Comprehensive statistics and visualizations are provided as well in the form of HTML reports, site profiles, and logos. In addition, the short run times allow for on-the-fly model training to quickly test hypotheses regarding data set, parameter, or feature choices.

## Methods

### The RNAProt framework

**Figure 1:**
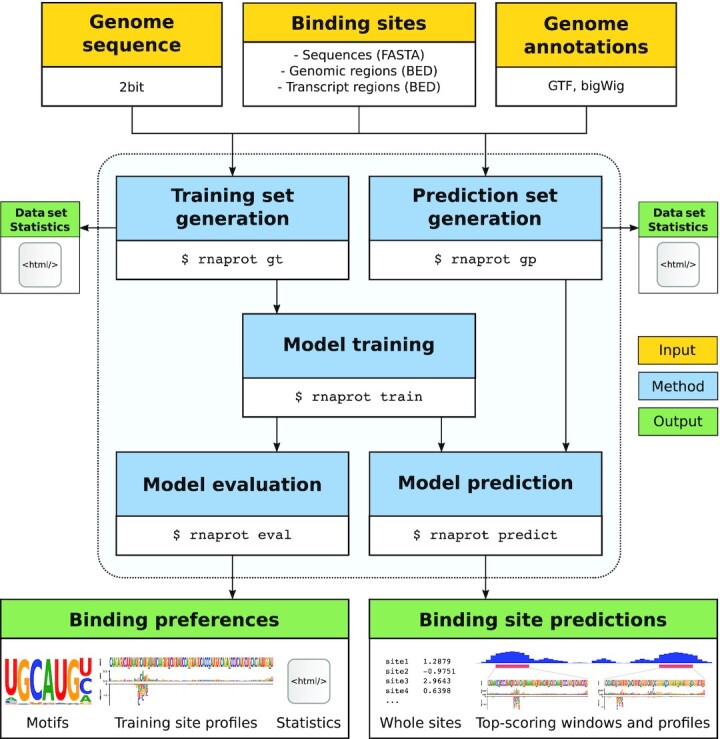
Overview of the RNAProt framework. The yellow boxes mark necessary framework inputs, the blue boxes mark the 5 program modes of RNAProt, and the green boxes mark the framework outputs. Arrows show the dependencies between inputs, modes, and outputs.

RNAProt utilizes RBP binding sites identified by CLIP-seq and related protocols to train an RNN-based model, which is then used to predict new binding sites on given input RNA sequences. Fig. 1 illustrates the RNAProt framework and its general workflow. RNAProt accepts RBP binding sites in FASTA or Browser Extensible Data (BED) formats. The latter also requires a genomic sequence file (.2bit format) and a genomic annotations file (Gene Transfer Format (GTF)).Compared to FASTA, genomes in binary 2bit format occupy less disk space, allow for faster sequence extraction, and also store repeat region information, which can be used as a feature. Binding sites can be supplied either as sequences, genomic regions, or aranscript regions (GTF file with corresponding transcript annotation required). Additional inputs are available depending on the binding site input type, as well as the selected features (see the “Supported features" section).

RNAProt can be run in 5 different program modes: generation of training and prediction sets, model training and evaluation, and model prediction (see the “Program modes" section). Depending on the executed mode, various output files are generated. For the data set generation modes, HTML reports can be output, which contain detailed statistics and visualizations regarding the positive, negative, or test data set. This way, for example, one can easily compare the positive input set with the generated negative set and spot possible similarities and differences. Reports include statistics on: site lengths, sequence complexity, di-nucleotide distributions, k-mer statistics, target region biotype, and overlap statistics, as well as additional statistics and visualizations for each selected feature. In the model evaluation mode, sequence and additional feature logos are output, as well as training site profiles for a subset of training sites to illustrate binding preferences. In the model prediction mode, whole site or moving window predictions are supported. In case of moving windows, position-wise scoring profiles are calculated and peak regions and top-scoring windows are extracted from the profiles. For a complete and up-to-date description, please refer to the online documentation on GitHub [24].

### Model architecture

RNAProt features an RNN-based model for binary classification of input sequences, which can be further customized from the command line or optimized using state-of-the-art hyperparameter optimization by Bayesian Optimization and Hyperband (BOHB) [25]. RNN-based models are well suited to learn from linear sequence information: in particular to learn dependencies between near or distant parts in a given sequence. This has been demonstrated in a number of related tasks over the years, from natural language processing to the analysis of time-series data and biological sequences like DNA or RNA. The type of RNN network used by RNAProt can be adjusted (Long Short-Term Memory [LSTM] [26] or Gated Recurrent Unit [27]), as can the numbers of hidden and full connected layers and dimensions, use of bidirectional RNN, or an embedding layer instead of 1-hot encoding for the sequence feature. As the optimizer, RNAProt applies an improved version of the Adam optimizer, termed AdamW [28]. RNAProt’s default hyperparameter setting was used to generate all the results presented in this work: a batch size of 50, learning rate of 0.001, weight decay of 0.0005, RNN model type of Gated Recurrent Unit, number of RNN layers set as 1, RNN layer dimensions set at 32, number of fully connected layers set as 1, dropout rate of 0.5, and no sequence embedding.

### Program modes

RNAProt is logically split into 5 different program modes: training set generation (rnaprot gt), prediction set generation (rnaprot gp), model training (rnaprot train), model evaluation (rnaprot eval), and model prediction (rnaprot predict). Separating data set generation from training or prediction has the advantage that feature values of interest have to be calculated or extracted only once (e.g., secondary structure, conservation scores, region annotations). Since model training is fast, one can then quickly train several models to assess which features or settings in general work best and move on to predictions. In the following we briefly introduce the mode functionalities.

#### Training set generation

This mode (rnaprot gt) is used to generate a training data set from a given set of RBP binding sites, which can be sequences, genomic regions, or transcript regions. In case sequences (FASTA format) are given as input, negative training sequences can be supplied or generated by k-nucleotide shuffling of the positive input sequences. In case genomic or transcript regions (BED format) are given as input, negatives can be supplied or selected randomly from gene or transcript regions containing positive sites (i.e., RBP binding sites identified by CLIP-seq). In general, we recommend supplying BED regions, as this allows RNAProt to automatically generate a negative set by randomly sampling sites from the genome or transcriptome. By default, negative sites are sampled based on 2 criteria: (i) sampling only from gene regions covered by positive sites; and (ii) no overlap with any positive site. The number of generated negative sites can be further specified, as can regions from which not to extract them. Output site lengths can be of variable or fixed size, and various filtering options are available to filter the sites by score, sequence complexity, region, or length. Concerning site lengths, RNAProt can train and predict on sequences of variable length due to its solely RNN-based architecture. For CNN-based methods this is usually not the case (unless the method internally applies padding before training and predicting). To keep data sets compatible with other tools, RNAProt therefore offers both variable and fixed-size outputs. Depending on the input type (see Table 1), different additional features can be selected for annotating the positive and negative sites (see the “Supported features" section for more details). An HTML report can be generated, providing statistics and visualizations to compare the positive with the negative set. The whole training data set is stored in a folder that forms the main input to the model training mode.

**Table 1:** RNAProts’s 3 supported input types (sequences, genomic regions [Genomic], transcript regions [Transcript]) and the features available for them

	Input		
Feature	Sequences	Genomic	Transcript
structure	YES	YES	YES
conservation scores	NO	YES	YES
exon-intron regions	NO	YES	NO
transcript regions	NO	YES	YES
repeat regions	NO	YES	YES
user-defined	NO	YES	YES

#### Model training

After generating a training set, a model can be trained on the data set in model training mode (rnaprot train). By default, all features of the training set are used to train the model, but specific features can be selected as well. Cross-validation is supported to estimate generalization performance, as well as learning curve plots and hyperparameter optimization using BOHB [25]. Unless cross-validation is specified, a model is trained using the default hyperparameters (or if BOHB is enabled, using the optimized hyperparameters after BOHB has finished) and output data are stored in a new folder, which serves as input to the model evaluation and model prediction modes.

#### Model evaluation

This mode (rnaprot eval) is used to visualize binding preferences of the model trained with rnaprot train. Sequence and additional feature logos of various lengths can be output, as well as training site profiles for a user-defined subset of training sites (see “Visualization” section for more details).

#### Prediction set generation

The prediction set generation mode (rnaprot gp) resembles rnaprot gt but, instead of generating a training set containing positives and negatives, it generates a prediction set from a given set of sites or sequences. Note that the types of additional features that can be added to the prediction set are dictated by the types used to train the model. Its output folder forms the input of rnaprot predict.

#### Model prediction

Model prediction mode (rnaprot predict) is used to predict whole binding sites or peak regions and top-scoring windows from sliding window profiles for a given set of sequences, genomic sites, or transcript sites. The prediction data set needs to be generated by rnaprot gp beforehand, as does the model, which needs to be trained through rnaprot train. Profiles of top-scoring windows can also be plotted and the input sites on which to predict can be specified.

### Supported features

RNAProt supports the following position-wise features, which can be utilized for training and prediction in addition to the sequence feature: secondary structure information (structural element probabilities), conservation scores (phastCons and phyloP), exon-intron annotation, transcript region annotation, and repeat region annotation. In addition, it also accepts user-defined region features (categorical or numerical; see documentation on GitHub [24] for details and examples), which no other tool so far offers. Table 1 lists the features available for each binding site input type.

#### Secondary structure information

RNAProt can include position-wise structure information, encoded as unpaired probabilities for different loop contexts (probabilities for the nucleotide being paired or inside external, hairpin, internal, or multi loops). ViennaRNA’s RNAplfold [29] is used with its sliding window approach, with user-definable parameters (by default these are window size = 70, maximum base pair span length = 50, and probabilities for regions of length u = 3). Note that genomic or transcript input sites are automatically extended on both sides (by window size) to get the most accurate structure predictions. This important feature is also not offered by any related tool.

#### Conservation scores

RNAProt supports 2 scores measuring evolutionary conservation (phastCons and phyloP). Human conservation scores were downloaded from the University of California Santa Cruz (UCSC) Genome Browser website, using the phastCons and phyloP scores generated from multiple sequence alignments of 99 vertebrate genomes to the human genome (as described in the GitHub manual [24]). RNAProt accepts scores in .bigWig format. To assign conservation scores to transcript regions, transcript regions are first mapped to the genome using the provided GTF file.

#### Exon-intron annotation

Exon-intron annotation in the form of 1-hot encoded exon or intron labels can also be added. Labels are assigned to each input BED site position by overlapping the site with genomic exon regions using BEDTools [30]. To unambiguously assign labels, RNAProt by default uses the most prominent isoform for each gene. The most prominent isoform for each gene gets selected through hierarchical filtering of the transcript information present in the input GTF file (for the benchmark results we used the Ensembl Genes 99 GRCh38.p13 version): given that the transcript is part of the GENCODE basic gene set, RNAProt selects transcripts based on their transcript support level (highest priority) and by transcript length (longer isoform preferred). The extracted isoform exons are then used for region type assignment. Alternatively, all exons can be used for labeling. Note that this feature is only available for genomic regions, as it is not informative for transcript regions, which would contain only exon labels. A user-defined isoform list can also be supplied, substituting the list of most prominent isoforms for annotation. Regions not overlapping with introns or exons can also be labeled separately (instead of labeled as intron).

#### Transcript region annotation

Similarly to the exon-intron annotation, binding regions can be labeled based on their overlap with transcript regions. Labels are assigned based on untranslated region (UTR) or coding region (CDS) overlap (5’UTR, CDS, 3’UTR, None), by taking the isoform information in the input GTF file. Again, the list of most prominent isoforms is used for annotation or, alternatively, a list of user-defined isoforms can be used. Additional annotation options include start and stop codon or transcript and exon border labeling.

#### Repeat region annotation

Repeat region annotation can also be added analogously to other region type annotations. This information is derived directly from the genomic sequence file (in .2bit format, from the UCSC website), where repeat regions identified by RepeatMasker and Tandem Repeats Finder are stored in lowercase letters.

### Visualization

To better understand the sequence or additional feature preferences of a model, RNAProt can plot logos and whole-site profiles. Both show position-wise features for each position, and the profile plots also include a saliency map track, plus a track that visualizes the effects of single-position mutations (also known as *in silico* mutagenesis) on the whole site score. Saliency maps visualize the gradient with respect to the input for each sequence position, thus showing the importance the trained model attributes to each sequence position and also its influence on the network output [31]. In contrast, *in silico* mutagenesis treats the network as a black box, where the input sequence is mutated at each position (3 mutations possible at each position, since there are 3 non-wild-type nucleotides) and the mutated sequences are scored by the network. For example, given a sequence AC, the mutated sequences would be CC, GC, UC, AA, AG, and AU. For a sequence of length *n*, we thus need to generate 3*n mutated sequences for which to calculate scores. The score difference (mutated sequence score minus wild-type sequence score) is then plotted for each mutated nucleotide at each position, with the height of the nucleotide corresponding to the score difference. This difference can be positive (i.e., the mutation increases the whole-site score) or negative (i.e., the mutation decreases the whole-site score). This way, both visualizations help in understanding what parts in a given sequence the model regards as important.

To generate the logo, RNAProt extracts top saliency value positions from a specified number of top scoring sites, and extends them to a defined logo length. The extracted subsequences (weighted by saliency) are then converted into a weight matrix and plotted with Logomaker [32].

### Tool comparison

#### Benchmark sets

For the tool comparison, we constructed 2 different benchmark sets. The first consists of 23 different PAR-CLIP, iCLIP, and High-throughput sequencing of RNA isolated by CLIP (HITS-CLIP) data sets (20 different RBPs) extracted from the original GraphProt publication. The second includes 30 eCLIP data sets (30 different RBPs) extracted from the Encyclopedia of DNA elements (ENCODE) website, [33]. For the GraphProt data sets, we defined a maximum number of positive and negative sites (each 5,000), and randomly selected these numbers for larger data sets. This was done since run times for DeepCLIP and DeepRAM can become very long as the number of sites increases (see the "Run time comparison" section for more details). For the eCLIP data sets, we aimed for 6,000 to 10,000 positive sites per data set during preprocessing and filtering. All sites were length-normalized to 81 nucleotides (nt) due to the fixed-size input required by DeepRAM. To generate the negative sets, we used RNAProt, which can automatically generate a set of random negative sites for a given set of positive input sites (i.e., RBP binding sites identified by CLIP-seq). By default, RNAProt randomly selects negative sites based on 2 criteria: (i) negative sites are sampled from gene regions containing positive sites; and (ii) a negative site should not overlap with any positive site. This setting was used to create the benchmark sets. The same number of random negative and positive instances was used throughout the benchmarks. More details on data preprocessing and data set construction can be found in the Supplementary Methods. For the run time comparison, we recorded single model training run times. Here, we randomly selected 5,000 positive and 5,000 negatives sites from the eCLIP RBFOX2 set, all with lengths of 81 nt, and trained each method 3 times on this set.

#### Tool setup and performance measurement

DeepCLIP, GraphProt, and RNAProt were benchmarked using their default parameters. For DeepRAM, we used their best-performing network architecture k-mer embedding with single layer CNN and bidirectional LSTM (ECBLSTM). The area under the receiver operating curve (AUC) was used in combination with 10-fold cross-validation to estimate and compare model generalization performances for the first 3 tools. Since DeepRAM does not offer a 10-fold cross-validation setting, we compared it separately to RNAProt using a hold-out setting (1 split with 90% of data for training and 10% for testing). For DeepCLIP, we set patience (early stopping) to 20 epochs and the maximum number of epochs to 200, which corresponds to the setting used for most data sets in the original publication. For RNAProt, we set the patience to 30 and the maximum number of epochs to 200 in cross-validation, while for the hold-out comparison we increased patience to 50, since we found that smaller data sets can sometimes benefit from increased patience. For the run time comparison, both DeepCLIP and RNAProt were set to a patience of 20 and a maximum number of epochs of 200. To signify differences in 10-fold cross-validation performance between the 3 methods, we calculated *P*-values using the 2-sided Wilcoxon test in R (version 3.6.2) for each data set and method combination. For comparing window prediction performances, we used the F-score (also known as F1 score or harmonic mean of precision and recall).

#### Computing benchmark results

To compute the benchmark results, we used 2 different desktop PCs: an AMD Ryzen7-2700X (32 GB RAM, GeForce RTX 2070 8 GB) and a Intel i7-8700k (32 GB RAM, Geforce GTX 1060 GPU 6 GB), both with Ubuntu 18.04 LTS installed. Tool run times were measured using solely the Intel i7, running single-model training 3 times and recording run times. In general, we found that RNAProt runs fine on a PC with 8 GB RAM and no GPU with the data set sizes found in the benchmark set. However, even an average consumer-grade GPU like the GTX 1060 drastically reduces run times (see the “Run time comparison” section results) and is thus recommended for on-the-fly model training (specifically an Nvidia card with ≥ 4 GB GPU RAM).

## Results and discussion

Below, we demonstrate RNAProt’s state-of-the-art performance and show its run time efficiency. In particular, we compared it to 2 recent deep-learning methods (DeepCLIP [34] and DeepRAM [35]), as well as GraphProt. We chose the first 2 because both provide usage instructions and are easy to install. Moreover, both compare favorably with many other methods in the field in their respective papers. As a reference, we also included the popular classical machine learning method GraphProt. Furthermore, we illustrate that RNAProt’s built-in visualizations can uncover known RBP binding preferences, and show that additional built-in features can boost performance. Finally, we exemplify the benefits of including structure information by improving the binding site prediction quality of the stem loop binding RBP Roquin.

### Cross-validation comparison

We first compared RNAProt in a standard 10-fold cross-validation setting with GraphProt and DeepCLIP, on 2 different sets of RBP data sets. The first set consists of 30 eCLIP data sets from 30 different RBPs, while the second set consists of 23 data sets from 20 RBPs, generated by various CLIP-seq protocols (see the “Benchmark sets” section for data set details). GraphProt is a popular classical machine learning method that uses a graph kernel with a Support Vector Machine classifier, while DeepCLIP is a recent deep-learning method featuring a combination of CNN and bidirectional LSTM. All 3 tools were trained using only sequence features.

**Figure 2:**
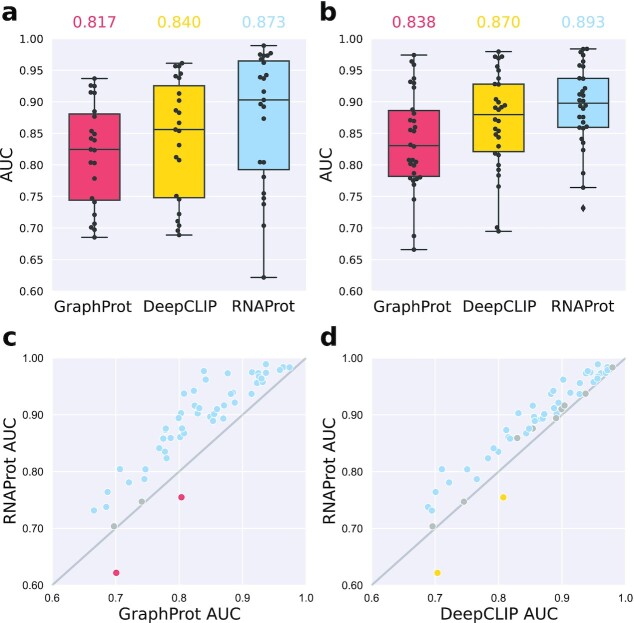
The 10-fold cross-validation results for GraphProt, DeepCLIP, and RNAProt. (a) Results for the first benchmark set contain 23 CLIP-seq data sets from 20 different RBPs and various CLIP-seq protocols (average method AUCs on top). (b) Results for the second benchmark set contain 30 eCLIP data sets from 30 different RBPs (average method AUCs on top). (c) Comparing single data set AUCs between GraphProt and RNAProt for all 53 data sets, the blue dots indicate a significantly better AUC for RNAProt (*n* = 49), the gray dots indicate no significant difference (*n* = 2), and the red dots indicate a significantly better AUC for GraphProt (*n* = 2). (d) Comparing single data set AUCs between DeepCLIP and RNAProt for all 53 data sets, the blue dots indicate a significantly better AUC for RNAProt (*n* = 42), the gray dots indicate no significant difference (*n* = 9), and the yellow dots indicate a significantly better AUC for DeepCLIP (*n* = 2). A 2-sided Wilcoxon test was used to calculate *P*-values (significance threshold = 0.05).

Fig. 2a and b show the 10-fold cross-validation results over the 2 benchmark sets for GraphProt, DeepCLIP, and RNAProt. For both sets, RNAProt achieves the highest total average AUC (87.26% and 89.30%), followed by DeepCLIP (84.03% and 87.00%), and GraphProt (81.71% and 83.81%). We note that both deep-learning methods outperform GraphProt on both sets. To signify performance differences between 2 methods, we calculated the 2-sided Wilcoxon test on the AUC distributions for each method combination and each of the 53 data sets (see Supplementary Tables S3 and S4 for AUCs and *P*-values). Fig. 2c and d contrast the single data set AUCs of GraphProt with RNAProt (Fig. 2c) and DeepCLIP with RNAProt (Fig. 2d), coloring significantly better method AUCs (GraphProt: red; DeepCLIP: yellow; RNAProt: blue). We can see that RNAProt outperforms GraphProt in 49 cases and DeepCLIP in 42 cases, while DeepCLIP and GraphProt both only perform better on 2 data sets. The 2 data sets are the same for both methods (ALKBH5, C17ORF85), which are from the original GraphProt publication. We can only speculate here that RNAProt’s lower performance might be due to some intrinsic incompatibilities of the data set and the utilized RNN network. As for the largely lower performances of DeepCLIP, we assume that it is possible to tune its hyperparameters (e.g., CNN filter or regularization settings) to increase its performance. Out of the box, however, RNAProt clearly outperforms DeepCLIP. Moreover, DeepCLIP has a clear disadvantage regarding run time (see the “Run time comparison” section below).

### Hold-out validation comparison

We also compared results to DeepRAM, a tool which allows the testing of various deep neural network architectures to compare their performances on DNA or RNA sequence data derived from chromatin immunoprecipitation with high-throughput sequencing (ChIP-seq) or CLIP-seq. For the comparison, we chose their best-performing architecture (ECBLSTM), a Word2Vec embedding of the input sequence (k-mer length = 3, stride = 1), followed by 1 CNN layer and 1 bidirectional LSTM layer. Since DeepRAM does not support cross-validation, we used a hold-out setting (i.e., 1 train-test split) for comparison, where models were trained on 90% of the data and tested on the remaining 10% for each data set. Note that we ran RNAProt with default hyperparameters, while DeepRAM does not offer default hyperparameters and requires hyperparameter optimization for each training run. We therefore manually reduced the number of random search iterations from 40 to 20 inside the DeepRAM code, to make the comparison more fair and run times more bearable. By this, the run time for a data set with 10,000 instances (81 nt long) got reduced to 5–6 hours, while for the same set RNAProt needs 1–2 minutes.

**Figure 3:**
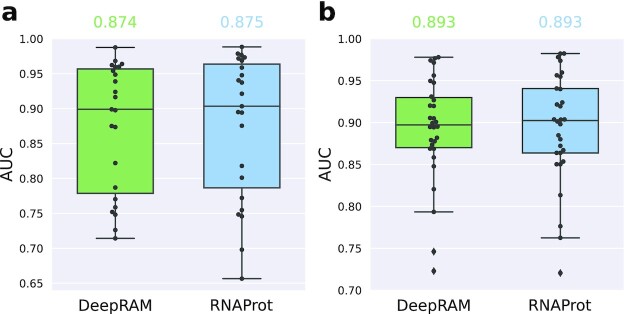
Hold-out validation results for DeepRAM and RNAProt. (a) Results for the first benchmark set contain 23 CLIP-seq data sets from 20 different RBPs and various CLIP-seq protocols. (b) Results for the second benchmark set contain 30 eCLIP data sets from 30 different RBPs. For both sets, we also report the average method AUC on top.

Fig. 3 shows the hold-out results over the 2 benchmark sets for DeepRAM and RNAProt. As we can see, average hold-out AUC performances of the 2 methods are very close for the 2 sets (DeepRAM: 87.42% and 89.28%; RNAProt: 87.50% and 89.34%). Again, there are only 2 data sets (ALKBH5, C17ORF85) where RNAProt performance drops considerably compared to DeepRAM, consistent with the cross-validation results above. For the remaining 51 data sets, there can be differences of 2% to 3% (both ways) but in general the performance is very similar (for full results, see Supplementary Tables S5 and S6). We thus can conclude that for the given data sets, there is no real advantage of using a more complex architecture like DeepRAM’s ECBLSTM.

As shown in the DeepRAM paper, more complex architectures like ECBLSTM can benefit from larger data sets (>10,000 positive instances). As our benchmark data sets contain between 1,338 and 9,206 positive sites (on average 6,389.4), ECBLSTM might perform better as data set sizes increase. However, >10,000 sites is often not a realistic estimate of the real number of RBP binding sites coming from a CLIP-seq experiment. For example, in order to get a high-confidence set of RBP binding sites from an eCLIP data set, the ENCODE consortium advises use of a strict filtering routine [36], leaving often only a few thousand sites, if not less, for subsequent analysis and model training. In addition, as pointed out in the DeepRAM paper, more complex models tend to be harder to interpret. On top of that, high test set performance does not guarantee that the model learned something biologically meaningful. We are also facing a trade-off between accuracy, interpretability, and run time. Depending on the application, the user might prefer a faster or a more accurate method, or they might care more about the interpretation of the prediction. In this regard, it would be interesting to explore in future studies whether ensemble predictions (including various more interpretable and more complex models) could help to combine individual model strengths.

### Run time comparison

Model training is known to be the computationally most expensive part of working with deep neural networks. We therefore compared the times it takes to train a single model with DeepCLIP, RNAProt, and, as a reference, the classical machine learning method GraphProt. Note that DeepRAM always runs a hyperparameter optimization for model training, making it unsuitable for this comparison. Specifically, we took 10,000 training instances (5,000 positives) of length 81 nt from the RBFOX2 eCLIP data set and trained a sequence model for all 3 methods (3 times each). We used default parameters for all methods, and for DeepCLIP and RNAProt set the patience and maximum number of epochs to 20 and 200, respectively (also see the “Computing benchmark results” section).

**Figure 4:**
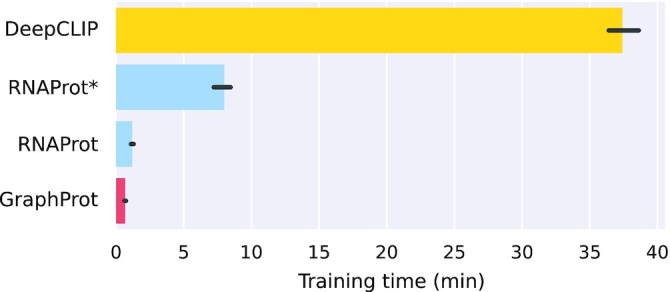
Model training time comparison. Training times are in minutes (averaged over 3 runs) for training a single model with 10,000 instances (81 nt) for GraphProt, RNAProt, and DeepCLIP. *RNAProt using CPU only for calculations (no GPU).

Fig. 4 shows the obtained average training times for DeepCLIP, RNAProt (CPU and GPU modes), and GraphProt (for full results, see Supplementary Table S7). We note that GraphProt model training is the fastest, at 40.3 seconds, followed by RNAProt (GPU) at 72 seconds, RNAProt (CPU) at 8 minutes, and DeepCLIP at 37.4 minutes. In other words, RNAProt GPU is 31 times faster (RNProt CPU 4.7 times faster) than DeepCLIP. This clearly shows RNAProt’s ability for on-the-fly model training, as well as the benefit of using a GPU (even an average consumer-grade GPU as described here). Since RNAProt supports many different features and settings, fast model training allows the user to try different settings for a specific task in a short amount of time. As for the run time difference, it seems that DeepCLIP currently does not support GPU computing, or at least we could not find any hints in the code. This would explain the slow run time, which unfortunately makes it less useful for on-the-fly training and testing. Still, its run times are much more practical than the ones we got with DeepRAM: due to its hard-coded hyperparameter optimization, DeepRAM can easily take 12 hours for model training (with the default number of random search iterations and benchmark data set sizes), even though it uses GPU computing.

### Visualizations capture known binding preferences

As deep-learning models are complex by design and thus hard to interpret, the development of visualizations that help to explain what is learned by a model is an important and active area of research. For RNAProt, we chose to visualize position-wise importances using 2 approaches: saliency maps and *in silico* mutagenesis (see the “Visualization” section for details).

**Figure 5:**
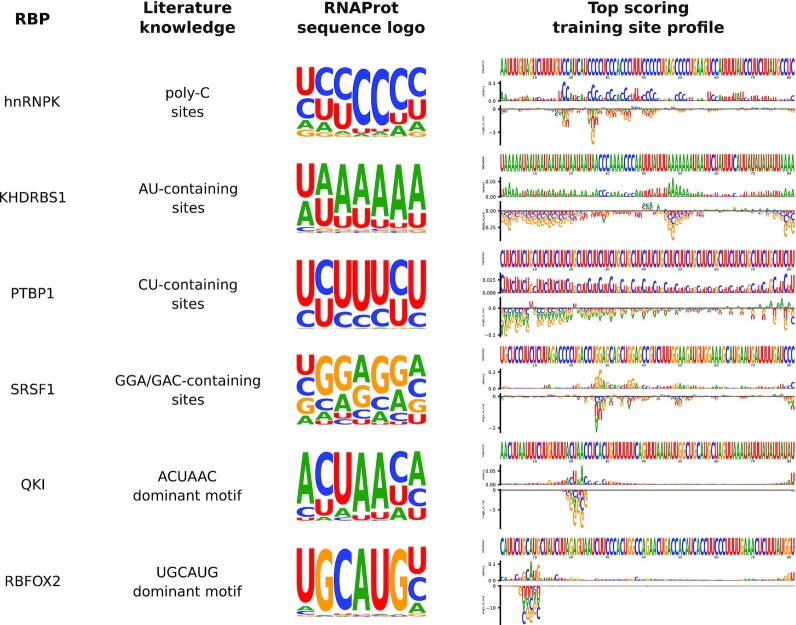
Comparison of RNAProt sequence logos and profiles with known RBP binding preferences. Literature knowledge was obtained from the ATtRACT database [37]. All models were trained using only the sequence feature. Logos were generated by extracting the top site saliency positions for each of the top 200 scoring training sites, and extending them by 3 on each side to generate logos of length 7. Logo character heights correspond to their respective saliency values at each of the 7 positions. On the right site, profiles for the top scoring training sites are shown, offering several tracks: the site nucleotide sequence, the position-wise saliency, and single mutation effects. The single mutations track shows how much every possible single nucleotide mutation at each position changes the total site score (positive or negative).

To compare RNAProt sequence logos and profiles with known RBP binding preferences from the literature, we trained sequence models on 6 different RBP data sets with known binding preferences. Fig. 5 shows the obtained sequence logo and known preferences (based on RBP motifs listed in the ATtRACT database [37]), as well as the top scoring training site profile for each RBP. As we can see, the logos clearly capture the literature preferences, both for RBPs without a single dominant motif (hnRNPK, KHDRBS1, PTBP1, SRSF1) and for RBPs with strong individual motifs (QKI, RBFOX2). This shows that saliency can be used to extract meaningful logos, which provide a rough idea about global model preferences. In addition, the saliency and mutation tracks give clues to local position-wise preferences. As shown, both match literature knowledge, but can also give interesting new insights. For example, important positions for the first 3 RBPs are more scattered in the observed profiles, while for QKI and RBFOX2 the model pays much more attention to the precise binding motif locations, with other positions having little effect on the model prediction. Both tracks are thus helpful to understanding local model decisions, but they are only informative for individual sites. To better understand global model preferences, we hope to integrate new visualizations in the near future, since this is also a very active area of research, albeit less mature than work on local preferences [38].

### Additional features boost performance

Since RNAProt supports various additional features on top of the sequence information, we also checked how including these features in training influences model performance. When generating training sets with RNAProt, the user can specify which features to compute and then, for training, can select which feature information the model should be trained on (see the “Supported features” section for details). For the comparison, we used RNA secondary structure, phastCons conservation scores, phyloP conservation scores, exon-intron annotation, and a combination of exon-intron and conservation scores.

**Figure 6:**
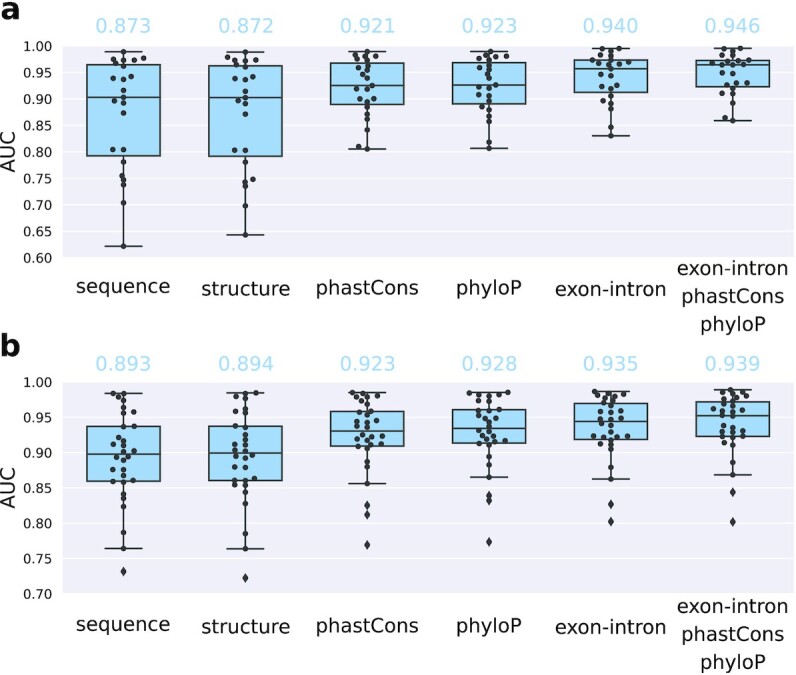
The 10-fold cross-validation results for RNAProt models trained with additional features. (a) Results for the first benchmark set contain 23 CLIP-seq data sets from 20 different RBPs and various CLIP-seq protocols. (b) Results for the second benchmark set contain 30 eCLIP data sets from 30 different RBPs. The “sequence” is included for reference, using only sequence information for training. For both sets, we report the average AUC with included additional feature(s) on top.

Fig. 6 shows the 10-fold cross-validation results for the 2 benchmark sets, for each described feature. We observe that the conservation and exon-intron features can, depending on the data set, strongly boost model performance on the benchmark sets. As for the structure feature, individual data set performances are usually very similar between structure and sequence-only models (see Supplementary Tables S1 and S2 for full results), although for the eCLIP set the overall performance with structure is slightly higher (89.41% vs 89.30% for the sequence-only model). We assume that this can be further tuned on the data set level by changing the structure calculation settings of RNAProt (different modes available, plus RNAplfold settings for window length, maximum base pair span, and mean probability region length). As for region type and conservation features, these performances of course highly depend on the selected negative regions. For example, using exon-intron annotations with negative regions located only inside introns and positive regions with a high amount of exonic sites will naturally lead to higher performance. But this does not make the model more useful. Thus, what the focus of the prediction should be is important. If the prediction should be on transcripts only, then exon-intron distinction becomes meaningless. However, some intrinsic bias of an RBP regarding regions can also be natural and of interest, such as when predicting on gene sequences containing introns and exons. In this regard, RNAProt offers several options to control negatives selection: users can either supply their own negative regions or the sampling of negative regions can be further specified by excluding certain genomic or transcript regions (see documentation for details).

Regarding the tested features, note that we did not include transcript or repeat region annotations in the comparison. As for the first feature, our tests showed performances similar to exon-intron inclusion, but we think that this feature needs an accurate (i.e., condition-specific) CDS and UTR region annotation to make sense. In line with this, it has been shown that context choice (i.e., selecting the authentic transcript or genomic context surrounding binding sites) affects the performances of RBP binding site prediction tools [39]. As RNAProt supports both genomic and transcript region annotations, it can easily be combined with isoform detection tools in future workflows. Regarding repeat region annotations, it did not make sense to test this feature since the eCLIP pipeline that produced the benchmark set binding sites only considers uniquely mapped reads. However, a recent pipeline update [36] now also allows mapping to certain repeat elements and has already led to the discovery of many new RBP binding sites overlapping with these elements. Repeat region annotation could thus become an informative feature once these data sets are available.

### Structure information can increase specificity

Given that additional features can increase predictive performance, we next checked whether they also can help in a more practical scenario. For this, we downloaded a data set consisting of predicted structurally conserved binding sites of the RBP Roquin (also termed constitutive decay elements [CDEs]) [40]. The CDEs were predicted using a biologically verified consensus structure consisting of a 6–8 bp long stem capped with a YRN (Y: C or U; R: A or G; N: any base) tri-nucleotide loop, including all human 3’UTRs as potential target regions. After preprocessing and training set generation (same number of random negatives; 81 nt site length), we trained a structure and a sequence model on the resulting 2,271 CDEs. For the structure prediction, we used an RNAplfold window length of 70 nt, a maximum base pair span of 50 nt, and a mean probability region length of 3 (see Supplementary Methods for more details).

Comparing the 10-fold cross-validation results of the 2 models, the sequence model achieves an average AUC of 79.22%, while the structure model performs almost 20% better (99.02%). We also note a high standard deviation for the individual sequence model AUC (7.66%), which is not the case for the structure model (0.43%). This means that the sequence model has problems with consistently classifying the test sites correctly, while the added structure information almost completely resolves this issue. We can thus conclude that the addition of structure information allows us to predict the given set of potential CDEs with high accuracy. As a reference, we also trained 2 GraphProt models (1 with sequence and 1 with structure information), which resulted in average AUCs of 70.81% and 78.49%, respectively.

**Figure 7:**
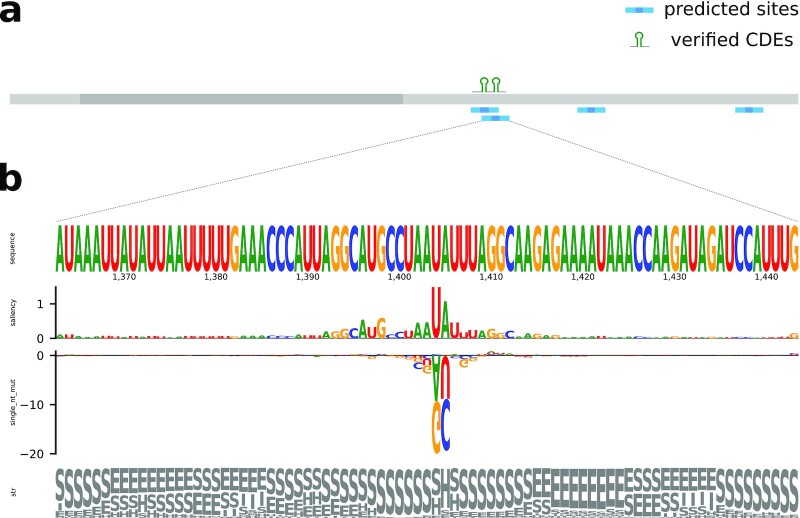
Roquin structure model predictions on the UCP3 gene transcript ENST00000314032.9. (a) The ENST00000314032.9 transcript (length 2,277 nt; 5' and 3' untranslated regions (UTRs) in light gray, coding sequence (CDS) in dark gray) is displayed together with verified and predicted Roquin binding sites (CDEs). (b) RNAProt site profile for the second verified CDE is shown with sequence, saliency map, single mutations, and structural elements tracks.

To complete the use case, the authors also experimentally verified 2 CDEs in the 3’UTR of the UCP3 gene (transcript ID ENST00000314032.9; length 2,277 nt). We therefore trained another structure model, excluding the 2 sites from the training set, and ran RNAProt using its window (profile) prediction mode on the transcript. Fig. 7a shows the transcript, along with verified and predicted CDEs. We note that our model predicts 4 CDEs in total (all in the 3’UTR), with 2 of them perfectly overlapping the verified CDEs. Fig. 7b shows the profile of the second site (compare to the red hairpin in Fig. 1C of Braun et al. [40]), with saliencies and the single mutations track highlighting the hairpin loop portion and parts of the surrounding stem. The stem loop can also be recognized in the structural elements track on the bottom. The single mutations track (measuring effects of single nucleotide changes on the whole-site score) indicates that the loop nucleotides are a particularly important sequence feature. In contrast, the structure feature contributes more to the area surrounding the loop, by providing the stem information. This again matches what is known about Roquin binding, with few sequence preferences in the hairpin aside from the described loop preferences. As a reference, we also trained a sequence model (validation set AUC 94.73%) and predicted CDEs on the transcript. This resulted in 18 predictions, with only 1 overlapping the first verified site, despite the very good validation AUC. This clearly demonstrates how additional features like structure information can help to make predictions more specific (F-score, sequence model = 0.10; F-score, structure model = 0.67).

## Conclusion

In this article we presented RNAProt, an RBP binding site prediction framework based on RNNs. Devised as an end-to-end method, RNAProt includes all necessary functionalities, from data set generation over model training to the evaluation of binding preferences and binding site prediction. We compared it to other popular tools in the field, showing its state-of-the-art performance and improved run time efficiency. The short training times allow for on-the-fly model training, which is great for quickly testing hypotheses regarding data set, parameter, or feature selections. Moreover, RNAProt is currently the most flexible method when it comes to supported position-wise features for learning, as well as input data types. RNAProt is easy to install and use, assisted by comprehensive documentation. Furthermore, it provides comprehensive statistics and visualizations, informing the user about data set characteristics and learned model properties. All this makes RNAProt a valuable tool to apply and include in RBP binding site analysis workflows.

## Availability of source code and requirements

- Project name: RNAProt
- Project page: https://github.com/BackofenLab/RNAProt
- Operating system(s): Linux
- Programming language: Python
- Other requirements: Anaconda
- Installation: conda install -c bioconda rnaprot
- License: MIT
- biotools ID: biotools:rnaprot
- RRID: SCR_021218

## Supplementary Material

giab054_GIGA-D-21-00105_Original_Submission

giab054_GIGA-D-21-00105_Revision_1

giab054_GIGA-D-21-00105_Revision_2

giab054_GIGA-D-21-00105_Revision_3

giab054_Response_to_Reviewer_Comments_Original_Submission

giab054_Response_to_Reviewer_Comments_Revision_1

giab054_Response_to_Reviewer_Comments_Revision_2

giab054_Reviewer_1_Report_Original_Submission Shiyong Liu -- 4/26/2021 Reviewed

giab054_Reviewer_1_Report_Revision_1 Shiyong Liu -- 5/26/2021 Reviewed

giab054_Reviewer_2_Report_Original_Submission Liangjiang Wang, Ph.D. -- 4/30/2021 Reviewed

giab054_Reviewer_3_Report_Original_Submission XIAOYONG Pan -- 5/1/2021 Reviewed

giab054_Reviewer_3_Report_Revision_1 XIAOYONG Pan -- 5/27/2021 Reviewed

giab054_Supplemental_Files

## Data Availability

All benchmark and training data sets used to create the reported results can be downloaded from Zenodo [41]. Supplementary Methods and Tables can be found on the *GigaScience* website and on GitHub [24]. A code snapshot as well as Supplementary Data are also available via GigaDB [42].

## Additional Files

**Supplementary Table S1:** 10-fold cross validation results for GraphProt, DeepCLIP, RNAProt, and RNAProt with additional features. Results for the first benchmark set, containing 23 CLIP-seq datasets from 20 different RBPs and various CLIP-seq protocols.

**Supplementary Table S2:** 10-fold cross validation results for GraphProt, DeepCLIP, RNAProt, and RNAProt with additional features. Results for the second benchmark set, containing 30 eCLIP datasets from 30 different RBPs.

**Supplementary Table S3:** 10-fold cross validation single fold AUC results for GraphProt, DeepCLIP, and RNAProt. Benchmark Set 1: Set from Table S1 (23 datasets). Benchmark Set 2: Set from Table S2 (30 datasets).

**Supplementary Table S4:** Two-sided Wilcoxon Test on Table S3 single fold AUCs, to determine significantly different AUCs between methods and single datasets. Calculated p-values for two method comparions are shown: RNAProt vs. GraphProt, and RNAProt vs. DeepCLIP.

**Supplementary Table S5:** Hold out validation results for DeepRAM and RNAProt. Results for the first benchmark set, containing 23 CLIP-seq datasets from 20 different RBPs and various CLIP-seq protocols.

**Supplementary Table S6:** Hold out validation results for DeepRAM and RNAProt. Results for the second benchmark set, containing 30 eCLIP datasets from 30 different RBPs.

**Supplementary Table S7:** Single model training runtime comparison for GraphProt, DeepCLIP, and RNAProt. Runtime is given in minutes (min), together with the mean runtime over three runs for each method.

**Supplementary methods**

Dataset construction

Cross validation comparison

Hold-out comparison

Roquin CDE dataset preparation and prediction

Runtime comparison

## List of abbreviations

AUC: area under the receiver operating curve; eCLIP: enhanced CLIP; CDE: constitutive decay element; CDS: coding region; CLIP-seq: cross-linking and immunoprecipitation followed by next-generation sequencing; CNN: convolutional neural network; iCLIP: individual-nucleotide resolution UV cross-linking and immunoprecipitation; LSTM: Long Short-Term Memory; PAR-CLIP: photoactivatable-ribonucleoside-enhanced crosslinking and immunoprecipitation; RBP: RNA-binding protein; RNN: Recurrent neural network; UTR: untranslated region.
