# Supplementary material for: RNAProt: an efficient and feature-rich RNA binding protein binding site predictor
Source: Gigascience. 2021 Aug 18;10(8):giab054. doi: 10.1093/gigascience/giab054 (PMC8372218; doi:10.1093/gigascience/giab054)
Supplement: giab054_GIGA-D-21-00105_Revision_3 [file giab054_giga-d-21-00105_revision_3.pdf]

GigaScience

RNAProt: An efficient and feature-rich RNA binding protein binding site predictor

--Manuscript Draft--

**Manuscript Number:**	GIGA-D-21-00105R3
**Full Title:**	RNAProt: An efficient and feature-rich RNA binding protein binding site predictor
**Article Type:**	Technical Note
**Funding Information:**	Deutsche Forschungsgemeinschaft (BA 2168/11-1 SPP 1738 and BA2168/11-2 SPP 1738) — Mr. Michael Uhl<br>Deutsche Forschungsgemeinschaft (BA 2168/3-3) — Dr. Van Dinh Tran<br>Deutsche Forschungsgemeinschaft (322977937/GRK2344 2017 MeInBio – BioInMe Research Training Group) — Mr. Florian Heyl<br>bundesministerium für bildung und forschung (RNAProNet-031L0164B) — Mr. Michael Uhl
**Abstract:**	Background:<br><br>CLIP-seq is the state-of-the-art technique to experimentally determine transcriptome-wide binding sites of RNA-binding proteins (RBPs). However, it relies on gene expression which can be highly variable between conditions, and thus cannot provide a complete picture of the RBP binding landscape. This creates a demand for computational methods to predict missing binding sites. Although there exist various methods using traditional machine learning and lately also deep learning, we encountered several problems: many of these are not well documented or maintained, making them difficult to install and use, or not even available. In addition, there can be efficiency issues, as well as little flexibility regarding options or supported features.<br>Results:<br><br>Here we present RNAProt, an efficient and feature-rich computational RBP binding site prediction framework based on recurrent neural networks (RNNs). We compare RNAProt with one traditional machine learning approach and two deep learning methods, demonstrating its state-of-the-art predictive performance, while at the same time offering better runtime efficiency. We further show that its implemented visualizations capture known binding preferences and thus can help to understand what is learned. Since RNAProt supports various additional features (including user-defined ones which no other tool offers), we also present their influence on benchmark set performance. Finally, we show the benefits of incorporating additional features, specifically structure information, when learning the binding sites of an hairpin loop binding RBP.<br>Conclusions:<br><br>RNAProt provides a complete framework for RBP binding site predictions, from dataset generation over model training to the evaluation of binding preferences and prediction. It offers state-of-the-art predictive performance as well as superior runtime efficiency, while at the same supporting more features and input types than any other tool available so far. RNAProt is easy to install and use, comes with a comprehensive documentation, and is accompanied by informative statistics and visualizations. All this makes RNAProt a valuable tool to apply in future RBP binding site research.
**Corresponding Author:**	Michael Uhl<br>University of Freiburg Faculty of Computer Technology: Albert-Ludwigs-Universitat Freiburg Technische Fakultat<br>Freiburg, GERMANY
**Corresponding Author Secondary Information:**	
**Corresponding Author's Institution:**	University of Freiburg Faculty of Computer Technology: Albert-Ludwigs-Universitat Freiburg Technische Fakultat

Powered by Editorial Manager® and ProduXion Manager® from Aries Systems Corporation

Corresponding Author's Secondary Institution:	
First Author:	Michael Uhl
First Author Secondary Information:	
Order of Authors:	Michael Uhl
	Van Dinh Tran
	Florian Heyl
	Rolf Backofen
Order of Authors Secondary Information:	
Response to Reviewers:	We added the Zenodo and GitHub citations, as well as the ORCID links on the title page.
Additional Information:	
**Question**	**Response**
Are you submitting this manuscript to a special series or article collection?	No
**Experimental design and statistics**<br><br>Full details of the experimental design and statistical methods used should be given in the Methods section, as detailed in our Minimum Standards Reporting Checklist. Information essential to interpreting the data presented should be made available in the figure legends.<br><br>Have you included all the information requested in your manuscript?	Yes
**Resources**<br><br>A description of all resources used, including antibodies, cell lines, animals and software tools, with enough information to allow them to be uniquely identified, should be included in the Methods section. Authors are strongly encouraged to cite Research Resource Identifiers (RRIDs) for antibodies, model organisms and tools, where possible.<br><br>Have you included the information requested as detailed in our Minimum Standards Reporting Checklist?	Yes

**Availability of data and materials**<br><br>All datasets and code on which the conclusions of the paper rely must be either included in your submission or deposited in publicly available repositories (where available and ethically appropriate), referencing such data using a unique identifier in the references and in the "Availability of Data and Materials" section of your manuscript.<br><br>Have you have met the above requirement as detailed in our Minimum Standards Reporting Checklist?	Yes

Powered by Editorial Manager® and ProduXion Manager® from Aries Systems Corporation

Revised manuscript
Click here to access/download;Manuscript;revised_manuscript.pdf

OXFORD
(GIGA)^n SCIENCE

GigaScience, 2017, 1–11

doi: xx.xxxx/xxxx
Manuscript in Preparation
Technical Note

TECHNICAL NOTE

RNAProt: An efficient and feature-rich RNA binding protein binding site predictor

Michael Uhl [1], Van Dinh Tran [1,*], Florian Heyl [1] and Rolf Backofen [1, 2,*]

[1]Bioinformatics Group, Department of Computer Science, University of Freiburg, Germany and [2]Signalling Research Centre CIBSS, University of Freiburg, Germany

[*]Corresponding authors: dinh@informatik.uni-freiburg.de, backofen@informatik.uni-freiburg.de

Abstract

Background CLIP-seq is the state-of-the-art technique to experimentally determine transcriptome-wide binding sites of RNA-binding proteins (RBPs). However, it relies on gene expression which can be highly variable between conditions, and thus cannot provide a complete picture of the RBP binding landscape. This creates a demand for computational methods to predict missing binding sites. Although there exist various methods using traditional machine learning and lately also deep learning, we encountered several problems: many of these are not well documented or maintained, making them difficult to install and use, or not even available. In addition, there can be efficiency issues, as well as little flexibility regarding options or supported features.
Results Here we present RNAProt, an efficient and feature-rich computational RBP binding site prediction framework based on recurrent neural networks (RNNs). We compare RNAProt with one traditional machine learning approach and two deep learning methods, demonstrating its state-of-the-art predictive performance, while at the same time offering better runtime efficiency. We further show that its implemented visualizations capture known binding preferences and thus can help to understand what is learned. Since RNAProt supports various additional features (including user-defined ones which no other tool offers), we also present their influence on benchmark set performance. Finally, we show the benefits of incorporating additional features, specifically structure information, when learning the binding sites of an hairpin loop binding RBP.
Conclusions RNAProt provides a complete framework for RBP binding site predictions, from dataset generation over model training to the evaluation of binding preferences and prediction. It offers state-of-the-art predictive performance as well as superior runtime efficiency, while at the same supporting more features and input types than any other tool available so far. RNAProt is easy to install and use, comes with a comprehensive documentation, and is accompanied by informative statistics and visualizations. All this makes RNAProt a valuable tool to apply in future RBP binding site research.

Key words: CLIP-seq; eCLIP; RBP binding site prediction; deep learning; recurrent neural networks; visualization

Introduction

RNA-binding proteins (RBPs) regulate many vital steps in the RNA life cycle, such as splicing, transport, stability, and translation [1]. Recent studies suggest a total number of more than 2,000 human RBPs, including 100s of unconventional RBPs, i.e., RBPs lacking known RNA-binding domains [2, 3, 4]. Numerous RBPs have been implicated in diseases like cancer, neurodegeneration, and genetic disorders [5, 6, 7], urging the need to speed up their functional characterization and shed light on their complex cellular interplay.

An important step to understand RBP function is to iden-

Compiled on: July 20, 2021.
Draft manuscript prepared by the author.

tify the precise RBP binding locations on regulated RNAs. In this regard, CLIP-seq (cross-linking and immunoprecipitation followed by next generation sequencing) [8] together with its popular modifications PAR-CLIP [9], iCLIP [10], and eCLIP [11] has become the state-of-the-art technique to experimentally determine transcriptome-wide binding sites of RBPs. A CLIP-seq experiment for a specific RBP results in a library of reads bound and protected by the RBP, making it possible to deduce its binding sites by mapping the reads back to the respective reference genome or transcriptome. In practice, computational analysis of CLIP-seq data has to be adapted for each CLIP-seq protocol [12]. Within the analysis, arguably the most critical part is the process of peak calling, i.e., to infer RBP binding sites from the mapped read profiles. Among the many existing peak callers, some popular tools are Piranha [13], CLIPper [14], and PureCLIP [15].

While peak calling is essential to separate authentic binding sites from unspecific interactions and thus reduce the false positive rate, it cannot solve the problem of expression dependency. In order to detect RBP binding sites by CLIP-seq, the target RNA has to be expressed at a certain level in the experiment. Since gene expression naturally varies between conditions, CLIP-seq data cannot be used to make condition-independent binding assumptions on a transcriptome-wide scale. Doing so would only increase the false negative rate, i.e., marking all regions not covered by CLIP-seq reads as non-binding, while in fact one cannot tell due to the lack of expression information. Moreover, expression variation is especially high for lncRNAs, an abundant class of ncRNAs gaining more and more attention due to their diverse cellular roles [16]. It is therefore of great importance to infer RBP binding characteristics from CLIP-seq data in order to predict missing binding sites. To give an example, [17] investigated the role of the splicing factor PTBP1 in differential splicing of the tumor suppressor gene ANXA7 in glioblastoma. Despite strong biological evidence for PTBP1 directly binding ANXA7, no binding site was found in a publicly available CLIP-seq dataset for PTBP1. Instead, only a computational analysis was capable to detect and correctly localize the presence of potential binding sites which were then experimentally validated.

Over the years, many approaches for RBP binding site prediction have been presented, from simple sequence motif search to more sophisticated methods incorporating classical machine learning and lately also deep learning. Some popular earlier methods include RNAcontext [18] and GraphProt [19], which can both incorporate RNA sequence and structure information into their predictive models. While RNAcontext utilizes a sequence and structure motif model, GraphProt uses a graph kernel coupled with Support Vector Machine (SVM), showing improved performance over motif-based techniques. From 2015 on, various deep learning based methods have been proposed, starting with DeepBind [20], which uses sequence information to train a convolutional neural network (CNN). Subsequent methods largely built upon this methodology, often using CNNs in combination with recurrent neural networks (RNNs) [21]. Some of them also incorporate additional features, usually specializing on a specific feature such as structure, evolutionary conservation, or region type information, to demonstrate its benefits. While these methods can certainly provide state-of-the-art predictive performance, we encountered several issues: many lack proper documentation, are not maintained, or not even available, even though they are presented as prediction tools in the original papers. Moreover, efficiency in terms of runtime can be a problem, as well as restricted options regarding data processing, and in general only few supported features.

Here we present RNAProt, a computational RBP binding site prediction framework based on RNNs, which takes care of the

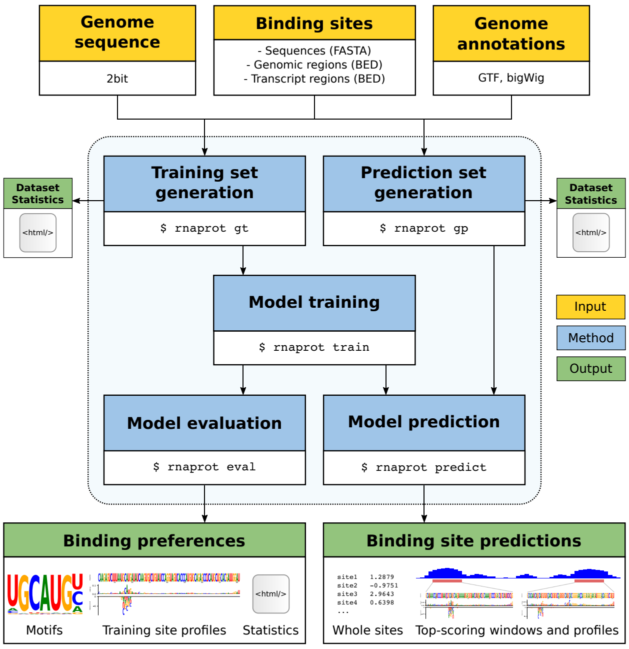

Figure 1. Overview of the RNAProt framework. Yellow boxes mark necessary framework inputs, blue boxes the five program modes of RNAProt, and green boxes the framework outputs. Arrows show the dependencies between inputs, modes, and outputs.

described issues: RNAProt provides both state-of-the-art performance and efficient runtimes. It comes with a comprehensive documentation and is easy to install via Conda. The availability of a Conda package, which to our knowledge no other related deep learning tool offers, also allows for an easy integration into larger workflows, such as Snakemake pipelines [22] or inside the Galaxy framework [23]. RNAProt offers various position-wise features on top of the sequence information, such as secondary structure, conservation scores, or region annotations, which can also be user-supplied ones. Through its use of an RNN-based architecture, RNAProt natively supports input sequences of variable length. In contrast, CNNs are constrained to fixed-sized inputs, which e.g. excludes the direct usage of variable-sized inputs usually defined by peak callers. Moreover, RNAProt is currently the most flexible method with regard to the support of input data types: apart from sequences and genomic regions, it can also handle transcript regions, providing automatic feature annotations for all three types. Comprehensive statistics and visualizations are provided as well, in the form of HTML reports, site profiles, and logos. In addition, the short runtimes allow for on-the-fly model training to quickly test hypotheses regarding dataset, parameter, or feature choices.

Methods

The RNAProt framework

RNAProt utilizes RBP binding sites identified by CLIP-seq and related protocols to train an RNN-based model, which is then used to predict new binding sites on given input RNA sequences. Figure 1 illustrates the RNAProt framework and its general workflow. RNAProt accepts RBP binding sites in FASTA or BED format. The latter one also requires a genomic sequence file (.2bit format) and a genomic annotations file (GTF format). Compared to FASTA, genomes in binary .2bit format occupy less disk space, allow for faster sequence extraction, and also store repeat region information, which can be used as a feature.

Binding sites can be supplied either as sequences, genomic regions, or as transcript regions (GTF file with corresponding transcript annotation required). Additional inputs are available, depending on the binding site input type as well as the selected features (see Supported Features Section).

RNAProt can be run in five different program modes: generation of training and prediction sets, model training and evaluation, and model prediction (see Program Modes section). Depending on the executed mode, various output files are generated. For the dataset generation modes, HTML reports can be output which contain detailed statistics and visualizations regarding the positive, negative, or test dataset. This way one can easily compare e.g. the positive input set with the generated negative set and spot possible similarities and differences. Reports include statistics on: site lengths, sequence complexity, di-nucleotide distributions, k-mer statistics, target region biotype and overlap statistics, as well as additional statistics and visualizations for each selected feature. In model evaluation mode, sequence and additional feature logos are output, as well as training site profiles for a subset of training sites to illustrate binding preferences. In model prediction mode, whole site or moving window predictions are supported. In case of moving windows, position-wise scoring profiles are calculated, and peak regions and top-scoring windows are extracted from the profiles. For a complete and up-to-date description, please refer to the online documentation on GitHub.

Model architecture

RNAProt features an RNN-based model for binary classification of input sequences, which can be further customized from the command line, or optimized using state-of-the-art hyperparameter optimization by BOHB [24]. RNN-based models are well suited to learn from linear sequence information, in particular to learn dependencies between near or distant parts in a given sequence. This has been demonstrated in a number of related tasks over the years, from natural language processing, to the analysis of time-series data and biological sequences like DNA or RNA. The type of RNN network used by RNAProt can be adjusted (Long Short-Term Memory (LSTM) [25] or Gated Recurrent Unit (GRU) [26]), as well as the number of hidden and full connected layers and dimensions, use of bidirectional RNN, or an embedding layer instead of one-hot encoding for the sequence feature. As optimizer, RNAProt applies an improved version of the Adam optimizer termed AdamW [27]. RNAProt's default hyperparameter setting was used to generate all the results presented in this work: batch size = 50, learning rate = 0.001, weight decay = 0.0005, RNN model type: GRU, number of RNN layers = 1, RNN layer dimensions 32, number of fully connected layers = 1, dropout rate = 0.5, and no sequence embedding.

Program modes

RNAProt is logically split into five different program modes: training set generation (`rnaprot gt`), prediction set generation (`rnaprot gp`), model training (`rnaprot train`), model evaluation (`rnaprot eval`), and model prediction (`rnaprot predict`). Separating dataset generation from training or prediction has the advantage that feature values of interest have to be calculated or extracted only once (e.g. secondary structure, conservation scores, region annotations). Since model training is fast, one can then quickly train several models to assess which features or settings in general work best and move on to predictions. In the following we briefly introduce the mode functionalities.

Training set generation

This mode (`rnaprot gt`) is used to generate a training dataset from a given set of RBP binding sites, which can be sequences, genomic regions, or transcript regions. In case sequences (FASTA format) are given as input, negative training sequences can be supplied or generated by k-nucleotide shuffling of the positive input sequences. In case genomic or transcript regions (BED format) are given as input, negatives can be supplied or selected randomly from gene or transcript regions containing positive sites (i.e., RBP binding sites identified by CLIP-seq). In general, we recommend to supply BED regions, as this allows RNAProt to automatically generate a negative set by randomly sampling sites from the genome or transcriptome. By default, negative sites are sampled based on two criteria: 1) sampling only from gene regions covered by positive sites 2) no overlap with any positive site. By default, negative sites are sampled based on two criteria: 1) sampling only from gene regions covered by positive sites 2) no overlap with any positive site. The number of generated negative sites can be further specified, as well as regions from which not to extract them. Output site lengths can be of variable or fixed size, and various filtering options are available to filter the sites by score, sequence complexity, region, or length. Concerning site lengths, RNAProt can train and predict on sequences of variable length due to its solely RNN-based architecture. For CNN-based methods this is usually not the case (unless the method internally applies padding before training and predicting). To keep datasets compatible with other tools, RNAProt therefore offers both variable and fixed-size outputs. Depending on the input type (see Table 1), different additional features can be selected for annotating the positive and negative sites (see Supported features section for more details). An HTML report can be generated, providing statistics and visualizations to compare the positive with the negative set. The whole training dataset is stored in a folder which forms the main input to the model training mode.

Model training

After generating a training set, a model can be trained on the dataset in model training mode (`rnaprot train`). By default, all features of the training set are used to train the model, but specific features can be selected as well. Cross validation is supported to estimate generalization performance, as well as learning curve plots, and hyperparameter optimization using BOHB [24]. Unless cross validation is specified, a model is trained using the default hyperparameters (or if BOHB is enabled using the optimized hyperparameters after BOHB has finished), and output in a new folder, which serves as input to the model evaluation and model prediction modes.

Model evaluation

This mode (`rnaprot eval`) is used to visualize binding preferences of the model trained with `rnaprot train`. Sequence and additional feature logos of various lengths can be output, as well as training site profiles for a user-defined subset of training sites (see Figure 5 and 7 for visualization examples, and Visualization section for more details).

Prediction set generation

The prediction set generation mode (`rnaprot gp`) resembles `rnaprot gt`, but instead of generating a training set containing positives and negatives, it generates a prediction set from a given set of sites or sequences to predict on. Note that the types of additional features that can be added to the prediction set are dictated by the types used to train the model. Its output folder forms the input of `rnaprot predict`.

Model prediction

Model prediction mode (`rnaprot predict`) is used to predict whole binding sites or peak regions and top-scoring windows from sliding window profiles for a given set of sequences, genomic sites, or transcript sites. The prediction dataset needs to be generated by `rnaprot gp` beforehand, as well as the model, which needs to be trained through `rnaprot train`. Profiles of top-scoring windows can also be plotted (see Figure 7b as example) and input sites on which to predict can be specified.

Supported features

RNAProt supports the following position-wise features that can be utilized for training and prediction in addition to the sequence feature: secondary structure information (structural element probabilities), conservation scores (phastCons and phyloP), exon-intron annotation, transcript region annotation, and repeat region annotation. In addition, it also accepts user-defined region features (categorical or numerical, see documentation for details and examples), which no other tool so far offers. Table 1 lists the features available for each binding site input type.

Table 1. RNAProts's three supported input types (sequences, genomic regions (Genomic), transcript regions (Transcript)) and the features available for them.

Feature	Sequences	Input Genomic	Transcript
structure	YES	YES	YES
conservation scores	NO	YES	YES
exon-intron regions	NO	YES	NO
transcript regions	NO	YES	YES
repeat regions	NO	YES	YES
user-defined	NO	YES	YES

Secondary structure information

RNAProt can include position-wise structure information encoded as unpaired probabilities for different loop contexts (probabilities for the nucleotide being paired or inside external, hairpin, internal or multi loops). ViennaRNA's RNAplfold is used with its sliding window approach, with user-definable parameters (by default these are window size = 70, maximum base pair span length = 50, and probabilities for regions of length u = 3). Note that genomic or transcript input sites are automatically extended on both sides (by window size) to get the most accurate structure predictions. This important feature is also not offered by any related tool.

Conservation scores

RNAProt supports two scores measuring evolutionary conservation (phastCons and phyloP). Human conservation scores were downloaded from the UCSC, using the phastCons and phyloP scores generated from multiple sequence alignments of 99 vertebrate genomes to the human genome (hg38, phastCons100way and phyloP100way datasets). RNAProt accepts scores in .bigWig format. To assign conservation scores to transcript regions, transcript regions are first mapped to the genome, using the provided GTF file.

Exon-intron annotation

Exon-intron annotation in the form of one-hot encoded exon or intron labels can also be added. Labels are assigned to each input BED site position by overlapping the site with genomic exon regions using bedtools. To unambiguously assign labels, RNAProt by default uses the most prominent isoform for each gene. The most prominent isoform for each gene gets selected through hierarchical filtering of the transcript information present in the input GTF file (for the benchmark results we used the Ensembl Genes 99 GRCh38.p13 version): given that the transcript is part of the GENCODE basic gene set, RNAProt selects transcripts based on their transcript support level (highest priority), and by transcript length (longer isoform preferred). The extracted isoform exons are then used for region type assignment. Alternatively, all exons can be used for labelling. Note that this feature is only available for genomic regions, as it is not informative for transcript regions, which would contain only exon labels. A user-defined isoform list can also be supplied, substituting the list of most prominent isoforms for annotation. Regions not overlapping with introns or exons can also be labeled separately (instead of labeled as intron).

Transcript region annotation

Similarly to the exon-intron annotation, binding regions can be labelled based on their overlap with transcript regions. Labels are assigned based on UTR or CDS region overlap (5'UTR, CDS, 3'UTR, None), by taking the isoform information in the input GTF file. Again the list of most prominent isoforms is used for annotation, or alternatively a list of user-defined isoforms. Additional annotation options include start and stop codon or transcript and exon border labelling.

Repeat region annotation

Repeat region annotation can also be added, analogously to other region type annotations. This information is derived directly from the genomic sequence file (in .2bit format, from the UCSC website), where repeat regions identified by RepeatMasker and Tandem Repeats Finder are stored in lowercase letters.

Visualization

To better understand the sequence or additional feature preferences of a model, RNAProt can plot logos and whole-site profiles. Both show position-wise features for each position, and the profile plots also include a saliency map track, plus a track that visualizes the effects of single position mutations (also known as *in silico* mutagenesis) on the whole site score. Saliency maps visualize the gradient with respect to the input for each sequence position, this way showing the importance the trained model attributes to each sequence position and thus also its influence on the network output [28]. In contrast, *in silico* mutagenesis treats the network as a black box, where the input sequence is mutated at each position (three mutations possible at each position since there are three non-wildtype nucleotides), and the mutated sequences are scored by the network. For example, given a sequence AC, the mutated sequences would be CC, GC, UC, AA, AG, and AU. For a sequence of length n we thus need to generate $3 * n$ mutated sequences to calculate scores for. The score difference (mutated sequence score minus wildtype sequence score) is then plotted for each mutated nucleotide at each position, with the height of the nucleotide corresponding to the score difference (see Figures 5 and 7 for examples). This difference can be positive (i.e., the mutation increases the whole-site score) or negative (i.e., the mutation decreases the whole-site score). This way, both visualizations help in understanding what parts the model regards as important in a given sequence.

To generate the logo, RNAProt extracts top saliency value positions from a specified number of top scoring sites, and extends them to a defined logo length. The extracted subsequences (weighted by saliency) are then converted into a weight matrix and plotted with Logomaker [29].

Tool comparison

Benchmark sets

For the tool comparison we constructed two different benchmark sets: The first one consists of 23 different PAR-CLIP, iCLIP, and HITS-CLIP datasets (20 different RBPs) extracted from the original GraphProt publication. The second one includes 30 eCLIP datasets (30 different RBPs) extracted from ENCODE. For the GraphProt datasets, we defined a maximum number of positive and negative sites (each 5,000), and randomly selected these numbers for larger datasets. This was done since runtimes for DeepCLIP and DeepRAM can become very long as the number of sites increases (see Runtime comparison for more details). For the eCLIP datasets, we aimed for 6,000 to 10,000 positive sites per dataset during preprocessing and filtering. All sites were length-normalized to 81 nt due to the fixed-size input required by DeepRAM. To generate the negative sets, we used RNAProt which can automatically generate a set of random negative sites for a given set of positive input sites (i.e., RBP binding sites identified by CLIP-seq). By default, RNAProt randomly selects negative sites based on two criteria: 1) negative sites are sampled from gene regions containing positive sites and 2) a negative site should not overlap with any positive site. This setting was used to create the benchmark sets. The same number of random negative and positive instances was used throughout the benchmarks. More details on data preprocessing and dataset construction can be found in the Supplementary Methods. For the runtime comparison, we recorded single model training runtimes. Here we randomly selected 5,000 positive and 5,000 negatives sites from the eCLIP RBFOX2 set, all with length 81 nt, and trained each method three times on this set.

Tool setup and performance measurement

DeepCLIP, GraphProt, and RNAProt were benchmarked using their default parameters. For DeepRAM we used their best performing network architecture (ECBLSTM). The area under the ROC curve (AUC) measure was used in combination with 10-fold cross validation to estimate and compare model generalization performances for the first three tools. Since DeepRAM does not offer a 10-fold cross validation setting, we compared it separately to RNAProt, using a hold-out setting (one split with 90% of data for training, 10% for testing). For DeepCLIP we set patience (early stopping) to 20 epochs and maximum number of epochs to 200, which corresponds to the setting used for most datasets in the original publication. For RNAProt, we set the patience to 30 and maximum number of epochs to 200 in cross validation, while for the hold-out comparison we increased patience to 50, since we found that smaller datasets can sometimes benefit from an increased patience. For the runtime comparison, both DeepCLIP and RNAProt were set to patience = 20 and maximum number of epochs = 200. To signify differences in 10-fold cross validation performance between the three methods, we calculated p-values using the two-sided Wilcoxon test in R (version 3.6.2), for each dataset and method combination. For comparing window prediction performances, we used the F-score (also known as F1 score or harmonic mean of precision and recall).

Computing benchmark results

To compute the benchmark results, we used two different desktop PCs: a AMD Ryzen7-2700X (32 GB RAM, GeForce RTX 2070 8 GB) and a Intel i7-8700k (32 GB RAM, Geforce GTX 1060 GPU 6 GB), both with Ubuntu 18.04 LTS installed. Tool runtimes were measured using solely the Intel i7, running single model training three times and recording runtimes. In general, we found that RNAProt runs fine on a PC with 8 GB RAM and no GPU with the dataset sizes found in the benchmark set.

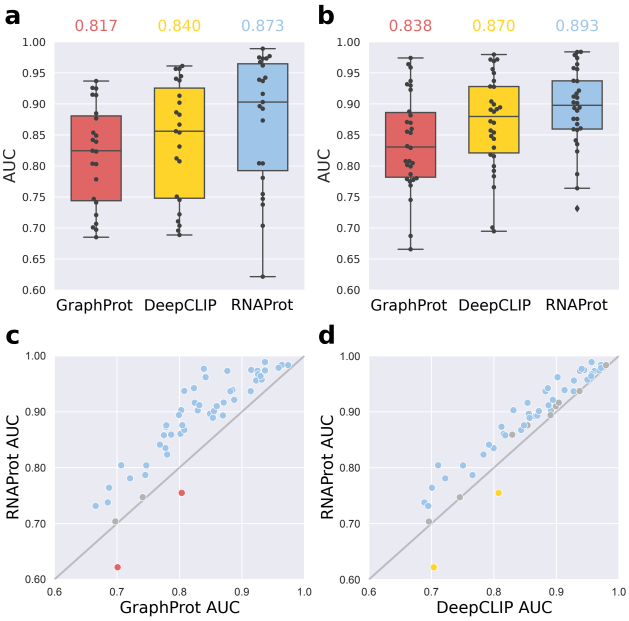

Figure 2. 10-fold cross validation results for GraphProt, DeepCLIP, and RNAProt. (a) Results for the first benchmark set, containing 23 CLIP-seq datasets from 20 different RBPs and various CLIP-seq protocols (average method AUCs op top). (b) Results for the second benchmark set, containing 30 eCLIP datasets from 30 different RBPs (average method AUCs on top). (c) Comparing single dataset AUCs between GraphProt and RNAProt, for all 53 datasets. Blue dots indicate a significantly better AUC for RNAProt (49), gray dots no significant difference (2), and red dots a significantly better AUC for GraphProt (2). (d) Comparing single dataset AUCs between DeepCLIP and RNAProt, for all 53 datasets. Blue dots indicate a significantly better AUC for RNAProt (42), gray dots no significant difference (9), and yellow dots a significantly better AUC for DeepCLIP (2). Two-sided Wilcoxon test was used to calculate p-values (significance threshold = 0.05).

However, even an average consumer-grade GPU like the GTX 1060 drastically reduces runtimes (see Runtime comparison results) and is thus recommended for on-the-fly model training (specifically an Nvidia card with $\geq$ 4 GB GPU RAM).

Results and Discussion

In the following we demonstrate RNAProt's state-of-the-art performance and show its runtime efficiency. In particular we compared to two recent deep learning methods (DeepCLIP [30] and DeepRAM [31]), as well as GraphProt. We chose the first two as both provide usage instructions and are easy to install. Moreover, both compare favorably with many other methods in the field in their respective papers. As a reference, we also included the popular classical machine learning method GraphProt. Furthermore, we illustrate that RNAProt's built-in visualizations can uncover known RBP binding preferences, and show that additional built-in features can boost performance. Finally, we exemplify the benefits of including structure information by improving the binding site prediction quality of the stem loop binding RBP Roquin.

Cross validation comparison

We first compared RNAProt in standard 10-fold cross validation setting with GraphProt and DeepCLIP, on two different sets of RBP datasets. The first set consists of 30 eCLIP datasets from 30 different RBPs, while the second one consists of 23 datasets from 20 RBPs, generated by various CLIP-seq protocols (see Benchmark sets section for dataset details). GraphProt is a popular classical machine learning method, using a graph kernel

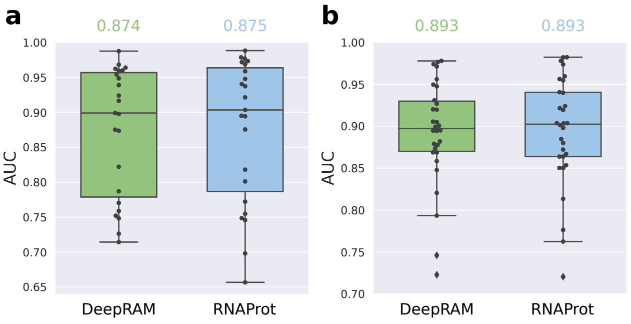

Figure 3. Hold-out validation results for DeepRAM and RNAProt. (a) Results for the first benchmark set, containing 23 CLIP-seq datasets from 20 different RBPs and various CLIP-seq protocols. (b) Results for the second benchmark set, containing 30 eCLIP datasets from 30 different RBPs. For both sets we also report the average method AUC on top.

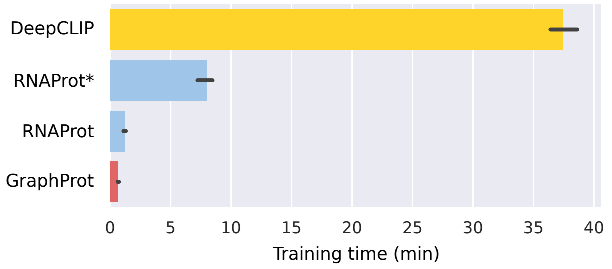

Figure 4. Model training time comparison. Training times in minutes (averaged over three runs) for training a single model with 10,000 instances (81 nt) for GraphProt, RNAProt, and DeepCLIP. * RNAProt using CPU only for calculations (no GPU).

with an SVM classifier, while DeepCLIP is a recent deep learning method featuring a combination of CNN and bidirectional LSTM. All three tools were trained using only sequences features.

Figure 2a and 2b show the 10-fold cross validation results over the two benchmark sets for GraphProt, DeepCLIP, and RNAProt. For both sets, RNAProt achieves the highest total average AUC (87.26%, 89.30%), followed by DeepCLIP (84.03%, 87.00%), and GraphProt (81.71%, 83.81%). We note that both deep learning methods outperform GraphProt on both sets. To signify performance differences between two methods, we calculated the two-sided Wilcoxon test on the AUC distributions for each method combination and each of the 53 datasets (see Supplementary Tables S3 and S4 for AUCs and p-values). Figure 2c and 2d contrast the single dataset AUCs of GraphProt with RNAProt (2c), and DeepCLIP with RNAProt (2d), coloring significantly better method AUCs (GraphProt: red, DeepCLIP: yellow, RNAProt: blue). We can see that RNAProt outperforms GraphProt in 49 and DeepCLIP in 42 cases, while DeepCLIP and GraphProt both only perform better on two datasets. The two datasets are the same for both methods (ALKBH5, C17ORF85), which are from the original GraphProt publication. We can only speculate here that RNAProt's lower performance might be due to some intrinsic incompatibilities of the dataset and the utilized RNN network. As for the largely lower performances of DeepCLIP, we assume that it is possible to tune its hyperparameters (e.g. CNN filter or regularization settings) to increase its performance. Out of the box however, RNAProt clearly outperforms DeepCLIP. Moreover, DeepCLIP has a clear disadvantage regarding runtime (see "Runtime comparison" below).

Hold-out validation comparison

We also compared to DeepRAM, a tool which allows the testing of various deep neural network architectures to compare their performances on DNA or RNA sequence data derived from ChIP-seq or CLIP-seq. For the comparison we chose their best performing architecture (ECBLSTM), i.e., a Word2Vec embedding of the input sequence (k-mer length = 3, stride = 1), followed by one CNN layer and one bidirectional LSTM layer. Since DeepRAM does not support cross validation, we used a hold-out setting (i.e., one train-test split) for comparison, where models were trained on 90% of the data and tested on the remaining 10% for each dataset. Note that we ran RNAProt with default hyperparameters, while DeepRAM does not offer default hyperparameters and requires hyperparameter optimization for each training run. We therefore manually reduced the number of random search iterations from 40 to 20 inside the DeepRAM code, to make the comparison more fair and runtimes more bearable. By this the runtime for a dataset with 10,000 instances (81 nt long) got reduced to 5 - 6 h, while for the same set RNAProt needs 1 - 2 min.

Figure 3 shows the hold-out results over the two benchmark sets for DeepRAM and RNAProt. As we can see, average hold-out AUC performances of the two methods are very close for the two sets (DeepRAM: 87.42% and 89.28% , RNAProt: 87.50% and 89.34%). Again, there are only two datasets (ALKBH5, C17ORF85) where RNAProt performance drops considerably compared to DeepRAM, consistent with the cross validation results above. For the remaining 51 datasets there can be differences of 2 to 3% (both ways), but in general the performance is very similar (for full results see Supplementary Tables S5 and S6). We thus can conclude that for the given datasets, there is no real advantage of using a more complex architecture like DeepRAM's ECBLSTM.

As shown in the DeepRAM paper, more complex architectures like ECBLSTM can benefit from larger datasets (> 10,000 positive instances). As our benchmark datasets contain between 1,338 and 9,206 positive sites (on average 6,389.4), ECBLSTM might perform better as dataset sizes increase. On the other hand, > 10,000 sites is often not a realistic estimate of the real number of RBP binding sites coming from a CLIP-seq experiment. For example, in order to get a high-confidence set of RBP binding sites from an eCLIP dataset, the ENCODE consortium advises to use a strict filtering routine [32], leaving often only a few thousand sites if not less for subsequent analysis and model training. In addition, as pointed out in the DeepRAM paper, more complex models tend to be harder to interpret. On top of that, high test set performance does not guarantee that the model learned something biologically meaningful. We are also facing a trade-off between accuracy, interpretability, and runtime. Depending on the application, the user might prefer a faster or a more accurate method, or he might care more about the interpretation of the prediction. In this regard, it would be interesting to explore in future studies whether ensemble predictions (including various more interpretable and more complex models) could help to combine individual model strengths.

Runtime comparison

Model training is known to be the computationally most expensive part when working with deep neural networks. We therefore compared the times it takes to train a single model with DeepCLIP, RNAProt, and as a reference the classical machine learning method GraphProt. Note that DeepRAM always runs a hyperparameter optimization for model training, making it unsuitable for this comparison. Specifically, we took 10,000 training instances (5,000 positives) of length 81 nt from the RBFOX2 eCLIP dataset and trained a sequence model for all three methods (three times each). We used default parameters for all

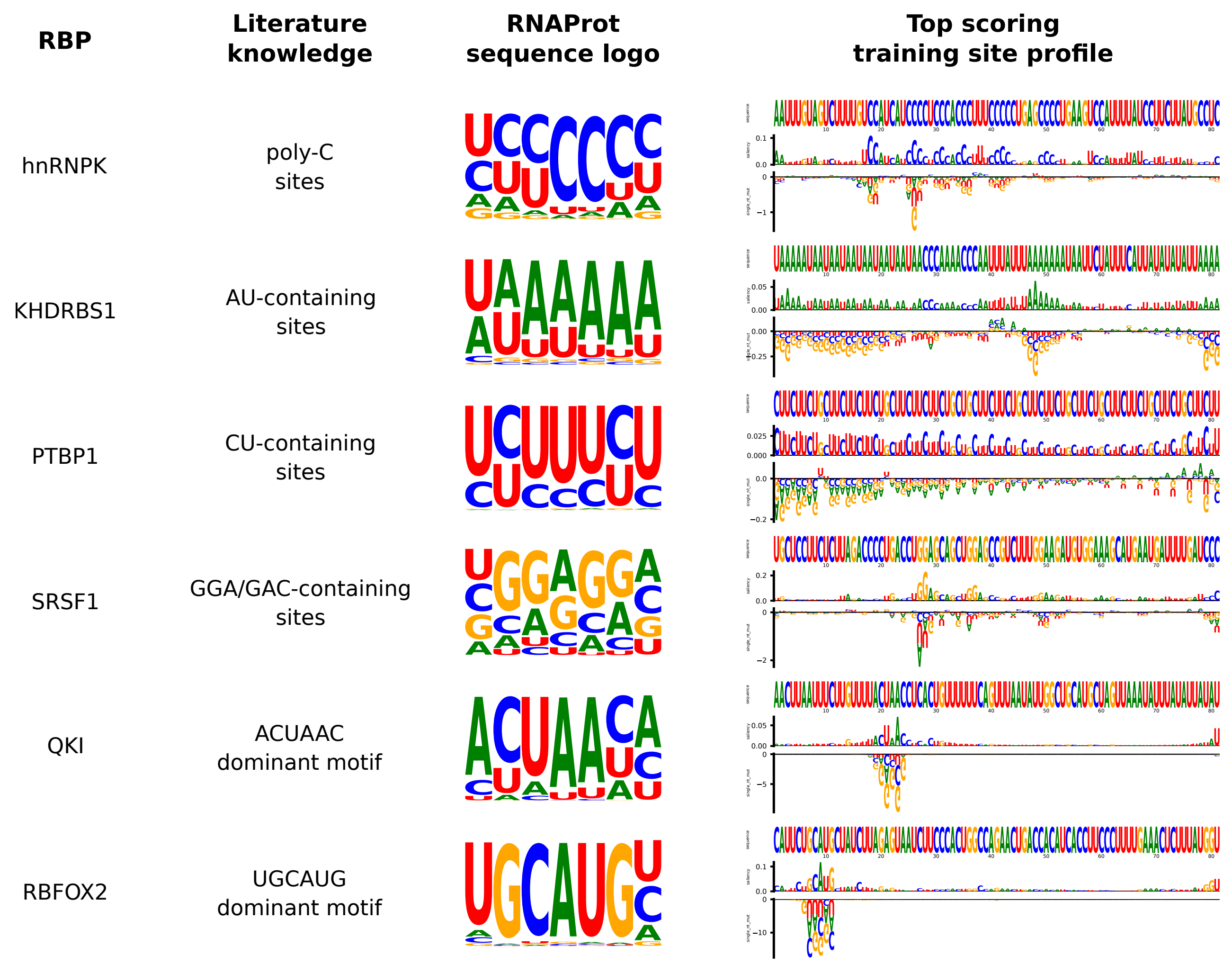

Figure 5. Comparison of RNAProt sequence logos and profiles with known RBP binding preferences. Literature knowledge was obtained from the ATtRACT database [33]. All models were trained using only the sequence feature. Logos were generated by extracting the top site saliency positions for each of the top 200 scoring training sites, and extending them by 3 on each side to generate logos of length 7. Logo character heights correspond to their respective saliency values at each of the 7 positions. On the right site profiles for the top scoring training sites are shown, offering several tracks: the site nucleotide sequence, the position-wise saliency, and single mutation effects. The single mutations track shows how much every possible single nucleotide mutation at each position changes the total site score (positive or negative).

methods, and for DeepCLIP and RNAProt set the patience and maximum number of epochs to 20 and 200 (also see "Computing benchmark results").

Figure 4 shows the obtained average training times for DeepCLIP, RNAProt (CPU and GPU mode), and GraphProt (for full results see Supplementary Table S7). We note that GraphProt model training is the fastest, with 40.3 seconds, followed by RNAProt (GPU) with 72 seconds, RNAProt (CPU) with 8 min, and DeepCLIP with 37.4 minutes. In other words, RNAProt GPU is 31 (RNProt CPU 4.7) times faster than DeepCLIP. This cleary shows RNAProt's ability for on-the-fly model training, as well as the benefit of using a GPU (even an average consumer-grade as described). Since RNAProt supports many different features and settings, fast model training allows the user to try different settings for a specific task in a short amount of time. As for the runtime difference, it seems that DeepCLIP currently does not support GPU computing, or at least we could not find any hints in the code. This would explain the slow runtime, which unfortunately makes it less useful for on-the-fly training and testing. Still, its runtimes are much more practical than the ones we got with DeepRAM, which due to the hard-coded hyperparameter optimization can easily take 12 h for model training (with default number of random search iterations and benchmark dataset sizes), even though it uses GPU computing.

Visualizations capture known binding preferences

As deep learning models are complex by design and thus hard to interpret, the development of visualizations that help to explain what is learned by a model is an important and active area of research. For RNAProt we chose to visualize position-wise importances using two approaches: saliency maps and *in silico* mutagenesis (see "Visualization" for details).

To compare RNAProt sequence logos and profiles with known RBP binding preferences from the literature, we trained sequence models on six different RBP datasets with known binding preferences. Figure 5 shows the obtained sequence logo and known preferences (based on RBP motifs listed in the ATtRACT database [33]), as well as the top scoring training site profile for each RBP. As we can see, the logos clearly capture the literature preferences, both for RBPs without a single dominant motif (hnRNPK, KHDRBS1, PTBP1, SRSF1), and for RBPs with strong individual motifs (QKI, RBFOX2). This shows that saliency can be used to extract meaningful logos, which provide a rough idea about global model preferences. In addition, the saliency and mutation tracks give clues on local position-wise preferences. As shown, both match literature knowledge, but can also give interesting new insights. For example, important positions for the first three RBPs are more scattered

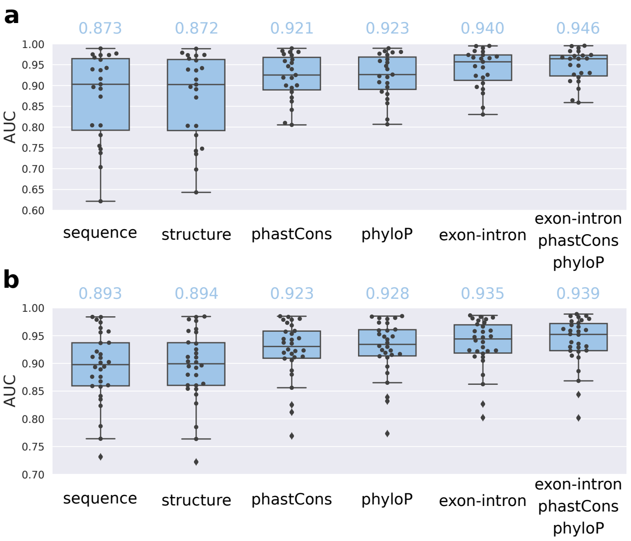

Figure 6. 10-fold cross validation results for RNAProt models trained with additional features. (a) Results for the first benchmark set, containing 23 CLIP-seq datasets from 20 different RBPs and various CLIP-seq protocols. (b) Results for the second benchmark set, containing 30 eCLIP datasets from 30 different RBPs. "sequence" is included for reference, using only sequence information for training. For both sets we report the average AUC with included additional feature(s) on top.

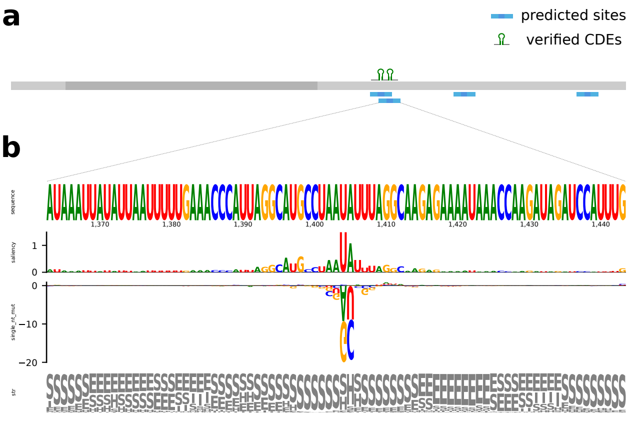

Figure 7. Roquin structure model predictions on the UCP3 gene transcript ENST00000314032.9. (a) ENST00000314032.9 transcript (length 2,277 nt, 5'UTR (light gray), CDS (dark gray), 3'UTR (light gray)) together with verified and predicted Roquin binding sites (CDEs). (b) RNAProt site profile for the second verified CDE. Shown tracks are: sequence, saliency map, single mutations track, structural elements track.

in the observed profiles, while for QKI and RBFOX2 the model pays much more attention to the precise binding motif locations, with other positions having little effect on the model prediction. Both tracks are thus helpful to understand local model decisions, but they are only informative for individual sites. To better understand global model preferences, we hope to integrate new visualizations in the near future, since this is, albeit less mature than work on local preferences, also a very active area of research [34].

Additional features boost performance

Since RNAProt supports various additional features on top of the sequence information, we also checked how including these features in training influences model performance. When generating training sets with RNAProt, the user can specify which features to compute, and then for training can select which feature information the model should be trained on (see "Supported features" for details). For the comparison we used RNA secondary structure, phastCons conservation scores, phyloP conservation scores, exon-intron annotation, and a combination of exon-intron and conservation scores.

Figure 6 shows the 10-fold cross validation results for the two benchmark sets, for each described feature. We observe that the conservation and exon-intron features can, depending on the dataset, strongly boost model performance on the benchmark sets. As for the structure feature, individual dataset performances are usually very similar between structure and sequence-only models (see Supplementary Table S1 and S2 for full results), although for the eCLIP set the overall performance with structure is slightly higher (89.41% vs 89.30%). We assume that this can be further tuned on the dataset level, by changing the structure calculation settings of RNAProt (different modes available plus RNAplfold settings for window length, maximum base pair span, and mean probability region length). As for region type and conservation features, these performances of course highly depend on the selected negative regions. For example, using exon-intron annotations with negative regions located only inside introns and positive regions with a high amount of exonic sites will naturally lead to a higher performance. But this does not make the model more useful. It is thus important what the focus of the prediction should be. If prediction should be on transcripts only, then exon-intron distinction becomes meaningless. However, some intrinsic bias of an RBP regarding regions can also be natural and of interest, e.g. when predicting on gene sequences containing introns and exons. In this regard, RNAProt offers several options to control negatives selection: users can either supply their own negative regions, or the sampling of negative regions can be further specified by excluding certain genomic or transcript regions (see documentation for details).

Regarding the tested features, note that we did not include transcript or repeat region annotations in the comparison. As for the first one, our tests showed performances similar to exon-intron inclusion, but we think that this feature needs an accurate (i.e., condition-specific) CDS and UTR region annotation to make sense. In line with this, it has been shown that context choice (i.e., selecting the authentic transcript or genomic context surrounding binding sites) affects the performances of RBP binding site prediction tools [35]. As RNAProt supports both genomic and transcript region annotations, it can easily be combined with isoform detection tools in future workflows. Regarding repeat region annotations, it did not make sense to test this feature since the eCLIP pipeline that produced the benchmark set binding sites only considers uniquely mapped reads. However, a recent pipeline update [32] now also allows mapping to certain repeat elements, and has already lead to the discovery of many new RBP binding sites overlapping with these elements. Repeat region annotation could thus become an informative feature once these datasets are available.

Structure information can increase specificity

Given that additional features can increase predictive performance, we next checked whether they also can help in a more practical scenario. For this we downloaded a dataset consisting of predicted structurally conserved binding sites of the RBP Roquin (also termed CDEs for constitutive decay elements) [36]. The CDEs were predicted using a biologically verified consensus structure consisting of a 6-8 bp long stem capped with a YRN (Y: C or U, R: A or G, N: any base) tri-nucleotide loop, including all human 3'UTRs as potential target regions. After preprocessing and training set generation (same number of random negatives, 81 nt site length), we trained a structure and a sequence

model on the resulting 2,271 CDEs. For the structure prediction we used an RNAplfold window length of 70 nt, a maximum base pair span of 50 nt, and a mean probability region length of 3 (see Supplementary Methods for more details).

Comparing the 10-fold cross validation results of the two models, the sequence model achieves an average AUC of 79.22%, while the structure model performs almost 20% better (99.02%). We also note a high standard deviation for the individual sequence model AUCs (7.66%), which is not the case for the structure model (0.43%). This means that the sequence model has problems with consistently classifying the test sites correctly, while the added structure information almost completely resolves this issue. We can thus conclude that the addition of structure information allows us to predict the given set of potential CDEs with high accuracy. As a reference, we also trained two GraphProt models (one with sequence, one with structure information), which resulted in average AUCs of 70.81% and 78.49%.

To complete the use case, the authors also experimentally verified two CDEs in the 3'UTR of the UCP3 gene (transcript ID ENST00000314032.9, length 2,277 nt). We therefore trained another structure model, excluding the two sites from the training set, and ran RNAProt using its window (profile) prediction mode on the transcript. Figure 7a shows the transcript along with verified and predicted CDEs. We note that our model predicts 4 CDEs in total (all in the 3'UTR), with two of them perfectly overlapping the verified CDEs. Figure 7b shows the profile of the second site (compare [36] Figure 1C, red hairpin), with saliencies and single mutations track highlighting the hairpin loop portion and parts of the surrounding stem. The stem loop can also be recognized in the structural elements track on the bottom. The single mutations track (measuring effects of single nucleotide changes on the whole-site score) indicates that the loop nucleotides are a particularly important sequence feature. In contrast, the structure feature contributes more to the loop surrounding, by providing the stem information. This again matches what is known about Roquin binding, having little sequence preferences in the hairpin, aside from the described loop preferences. As a reference, we also trained a sequence model (validation set AUC 94.73%) and predicted CDEs on the transcript. This resulted in 18 predictions, with only one overlapping the first verified site, despite the very good validation AUC. This clearly demonstrates how additional features like structure information can help to make predictions more specific (F-score sequence model = 0.10, F-score structure model = 0.67).

Conclusion

In this article we presented RNAProt, an RBP binding site prediction framework based on RNNs. Devised as an end-to-end method, RNAProt includes all necessary functionalities, from dataset generation over model training to the evaluation of binding preferences and binding site prediction. We compared it to other popular tools in the field, showing its state-of-the-art performance, while at the same time offering improved runtime efficiency. The short training times allow for on-the-fly model training, which is great for quickly testing hypotheses regarding dataset, parameter, or feature selections. Moreover, RNAProt is currently the most flexible method when it comes to supported position-wise features for learning, as well as input data types. RNAProt is easy to install and use, assisted by a comprehensive documentation. Furthermore, it provides comprehensive statistics and visualizations, informing the user about datatset characteristics and learned model properties. All this makes RNAProt a valuable tool to apply and include in RBP binding site analysis workflows.

Availability of source code and requirements

Lists the following:

- Project name: RNAProt
- Project page: `https://github.com/BackofenLab/RNAProt`
- Operating system(s): Linux
- Programming language: Python
- Other requirements: Anaconda
- Installation: `conda install -c bioconda rnaprot`
- License: MIT
- biotools ID: biotools:rnaprot
- RRID: SCR_021218

Availability of supporting data and materials

All benchmark and training datasets used to create the reported results can be downloaded from Zenodo [37]. Supplementary methods and tables can be found on GigaScience and on GitHub [38]. A code snapshot as well the supplementary data is also available on GigaDB [39].

Declarations

List of abbreviations

AUC: Area under the ROC curve; ECBLSTM: DeepRAM's best performing model setting; eCLIP: enhanced CLIP (CLIP-seq variant); CDE: Constitutive decay element (structured RNA region recognized by RBP Roquin); CDS: Coding region; CLIP-seq: Cross-linking and immunoprecipitationfollowed by next generation sequencing; CNN: Convolutional neural network; GRU: Gated recurrent unit; iCLIP: Individual-nucleotide resolution UV cross-linking and immunoprecipitation (CLIP-seq variant); LSTM: Long short-term memory; PAR-CLIP: Photoactivatable-ribonucleoside-enhanced crosslinking and immunoprecipitation (CLIP-seq variant); RBP: RNA-binding protein; RNN: Recurrent neural network; SVM: Support vector machine; UTR: Untranslated region.

Ethical Approval

Not applicable.

Consent for publication

Not applicable.

Competing Interests

The authors declare that they have no competing interests.

Funding

MU was funded by Deutsche Forschungsgemeinschaft (DFG) grant BA 2168/11-1 SPP 1738, BA2168/11-2 SPP 1738, and by the Bundesministerium für Bildung und Forschung (BMBF), RNAProNet-031L0164B. VDT was funded by DFG grant BA 2168/3-3. FH was funded by DFG grant 322977937/GRK2344 2017 MeInBio – BioInMe Research Training Group.

Author's Contributions

RB, VDT, and MU conceived the study. FH contributed Wilcoxon test p-values and DeepCLIP cross validation results. VDT and MU implemented the network part. MU performed the remaining data analysis, wrote the draft, and implemented the tool. All authors reviewed and approved the final manuscript.

Acknowledgements

We thank Martin Raden for his invaluable suggestions on the topic.

References

1. Gerstberger S, Hafner M, Tuschl T. A census of human RNA-binding proteins. Nature Reviews Genetics 2014;15(12):829.
2. Brannan KW, Jin W, Huelga SC, Banks CA, Gilmore JM, Florens L, et al. SONAR discovers RNA-binding proteins from analysis of large-scale protein-protein interactomes. Molecular cell 2016;64(2):282–293.
3. Hentze MW, Castello A, Schwarzl T, Preiss T. A brave new world of RNA-binding proteins. Nature Reviews Molecular Cell Biology 2018;19(5):327.
4. Liu L, Li T, Song G, He Q, Yin Y, Lu JY, et al. Insight into novel RNA-binding activities via large-scale analysis of lncRNA-bound proteome and IDH1-bound transcriptome. Nucleic acids research 2019;47(5):2244–2262.
5. Gerstberger S, Hafner M, Ascano M, Tuschl T. Evolutionary conservation and expression of human RNA-binding proteins and their role in human genetic disease. In: Systems biology of RNA binding proteins Springer; 2014.p. 1–55.
6. Pereira B, Billaud M, Almeida R. RNA-binding proteins in cancer: old players and new actors. Trends in cancer 2017;3(7):506–528.
7. Conlon EG, Manley JL. RNA-binding proteins in neurodegeneration: mechanisms in aggregate. Genes & development 2017;31(15):1509–1528.
8. Licatalosi DD, Mele A, Fak JJ, Ule J, Kayikci M, Chi SW, et al. HITS-CLIP yields genome-wide insights into brain alternative RNA processing. Nature 2008;456(7221):464.
9. Hafner M, Landthaler M, Burger L, Khorshid M, Hausser J, Berninger P, et al. Transcriptome-wide identification of RNA-binding protein and microRNA target sites by PAR-CLIP. Cell 2010;141(1):129–141.
10. König J, Zarnack K, Rot G, Curk T, Kayikci M, Zupan B, et al. iCLIP reveals the function of hnRNP particles in splicing at individual nucleotide resolution. Nature structural & molecular biology 2010;17(7):909.
11. Van Nostrand EL, Pratt GA, Shishkin AA, Gelboin-Burkhart C, Fang MY, Sundararaman B, et al. Robust transcriptome-wide discovery of RNA-binding protein binding sites with enhanced CLIP (eCLIP). Nature methods 2016;13(6):508.
12. Uhl M, Houwaart T, Corrado G, Wright PR, Backofen R. Computational analysis of CLIP-seq data. Methods 2017;118:60–72.
13. Uren PJ, Bahrami-Samani E, Burns SC, Qiao M, Karginov FV, Hodges E, et al. Site identification in high-throughput RNA-protein interaction data. Bioinformatics 2012;28(23):3013–3020.
14. Lovci MT, Ghanem D, Marr H, Arnold J, Gee S, Parra M, et al. Rbfox proteins regulate alternative mRNA splicing through evolutionarily conserved RNA bridges. Nature structural & molecular biology 2013;20:1434.
15. Krakau S, Richard H, Marsico A. PureCLIP: capturing target-specific protein-RNA interaction footprints from single-nucleotide CLIP-seq data. Genome biology 2017;18(1):240.
16. Kornienko AE, Dotter CP, Guenzl PM, Gisslinger H, Gisslinger B, Cleary C, et al. Long non-coding RNAs display higher natural expression variation than protein-coding genes in healthy humans. Genome biology 2016;17(1):14.
17. Ferrarese R, Harsh GR, Yadav AK, Bug E, Maticzka D, Reichardt W, et al. Lineage-specific splicing of a brain-enriched alternative exon promotes glioblastoma progression. The Journal of clinical investigation 2014;124(7):2861–2876.
18. Kazan H, Ray D, Chan ET, Hughes TR, Morris Q. RNAcontext: a new method for learning the sequence and structure binding preferences of RNA-binding proteins. PLoS computational biology 2010;6(7):e1000832.
19. Maticzka D, Lange SJ, Costa F, Backofen R. GraphProt: modeling binding preferences of RNA-binding proteins. Genome biology 2014;15(1):R17.
20. Alipanahi B, Delong A, Weirauch MT, Frey BJ. Predicting the sequence specificities of DNA-and RNA-binding proteins by deep learning. Nature biotechnology 2015;33(8):831.
21. Pan X, Yang Y, Xia CQ, Mirza AH, Shen HB. Recent methodology progress of deep learning for RNA-protein interaction prediction. Wiley Interdisciplinary Reviews: RNA 2019;p. e1544.
22. Mölder F, Jablonski KP, Letcher B, Hall MB, Tomkins-Tinch CH, Sochat V, et al. Sustainable data analysis with Snakemake. F1000Research 2021;10:33.
23. Afgan E, Baker D, Batut B, Van Den Beek M, Bouvier D, Čech M, et al. The Galaxy platform for accessible, reproducible and collaborative biomedical analyses: 2018 update. Nucleic acids research 2018;46(W1):W537–W544.
24. Falkner S, Klein A, Hutter F. BOHB: Robust and efficient hyperparameter optimization at scale. In: International Conference on Machine Learning PMLR; 2018. p. 1437–1446.
25. Hochreiter S, Schmidhuber J. Long short-term memory. Neural computation 1997;9(8):1735–1780.
26. Cho K, Van Merriënboer B, Bahdanau D, Bengio Y. On the properties of neural machine translation: Encoder-decoder approaches. arXiv preprint arXiv:14091259 2014;.
27. Loshchilov I, Hutter F. Decoupled weight decay regularization. arXiv preprint arXiv:171105101 2017;.
28. Li J, Chen X, Hovy E, Jurafsky D. Visualizing and understanding neural models in nlp. arXiv preprint arXiv:150601066 2015;.
29. Tareen A, Kinney JB. Logomaker: beautiful sequence logos in Python. Bioinformatics 2020;36(7):2272–2274.
30. Grønning AGB, Doktor TK, Larsen SJ, Petersen USS, Holm LL, Bruun GH, et al. DeepCLIP: predicting the effect of mutations on protein–RNA binding with deep learning. Nucleic acids research 2020;48(13):7099–7118.
31. Trabelsi A, Chaabane M, Ben-Hur A. Comprehensive evaluation of deep learning architectures for prediction of DNA/RNA sequence binding specificities. Bioinformatics 2019;35(14):i269–i277.
32. Van Nostrand EL, Pratt GA, Yee BA, Wheeler EC, Blue SM, Mueller J, et al. Principles of RNA processing from analysis of enhanced CLIP maps for 150 RNA binding proteins. Genome biology 2020;21:1–26.
33. Giudice G, Sánchez-Cabo F, Torroja C, Lara-Pezzi E. ATtRACT—a database of RNA-binding proteins and associated motifs. Database 2016;2016.
34. Koo PK, Ploenzke M. Deep learning for inferring transcription factor binding sites. Current opinion in systems biology 2020;.

35. Uhl M, Backofen R, et al. Improving CLIP-seq data analysis by incorporating transcript information. BMC genomics 2020;21(1):1–8.
36. Braun J, Fischer S, Xu ZZ, Sun H, Ghoneim DH, Gimbel AT, et al. Identification of new high affinity targets for Roquin based on structural conservation. Nucleic acids research 2018;46(22):12109–12125.
37. Uhl M. RNAProt: An efficient and feature-rich RNA binding protein binding site predictor. Zenodo 2021;https://doi.org/10.5281/zenodo.5083311.
38. Uhl M. RNAProt: An efficient and feature-rich RNA binding protein binding site predictor. GitHub repository 2021;https://github.com/BackofenLab/RNAProt.
39. Uhl M, Tran VD, Heyl F, Backofen R. Supporting data for "RNAProt: An efficient and feature-rich RNA binding protein binding site predictor". GigaScience Database 2021;http://dx.doi.org/10.5524/100916.

Supplementary methods

Click here to access/download
Supplementary Material
Supplementary_methods.pdf

Supplementary tables

Click here to access/download
Supplementary Material
Supplementary_tables_S1-S7.xlsx
