# Supplementary material for: RNAProt: an efficient and feature-rich RNA binding protein binding site predictor
Source: Gigascience. 2021 Aug 18;10(8):giab054. doi: 10.1093/gigascience/giab054 (PMC8372218; doi:10.1093/gigascience/giab054)
Supplement: giab054_Supplemental_Files [file giab054_supplemental_files.zip › Supplementary_methods.pdf]

RNAProt: An efficient and feature-rich RNA binding protein binding site predictor
Supplementary Material

Michael Uhl, Van Dinh Tran, Florian Heyl, and Rolf Backofen

April 2, 2021

Supplementary methods

Dataset construction

For the tool comparison we constructed two different benchmark sets: the first one includes 23 different PAR-CLIP, iCLIP, and HITS-CLIP datasets (20 different RBPs) extracted from the original GraphProt publication [3]. The second one consists of 30 eCLIP datasets (30 different RBPs) extracted from ENCODE.

For the first set, CLIP-Seq datasets used for benchmarking GraphProt were obtained from here. Sets for hyperparameter optimization and training were merged, hg19 genomic regions (corresponding to uppercase sequence parts, also termed viewpoint regions) were extracted from the FASTA headers, and lifted over to hg38, using the UCSC's liftOver command line tool. Viewpoint regions were filtered by a maximum length of 60 nt and extended to a new constant length of 81 nt, for CNN method compatibility. For each of the 24 datasets, we randomly selected a maximum of 5,000 positive and negative sites each, in order to keep model training times for DeepRAM [5] and DeepCLIP [2] reasonable. Note that we removed the PTB dataset from the benchmark set, as the sampled 10,000 sites showed to be non-informative (resulting AUCs of 50% for all methods). This led us to the final benchmark set size of 23 datasets.

For the eCLIP set we extracted data out of two cell lines (HepG2, K562) from ENCODE [4] (November 2018 release). We directly used the genomic binding regions (genome assembly GRCh38) identified by ENCODE's in-house peak caller CLIPper, which are available in BED format for each RBP and each replicate, often for both cell lines (thus 4 replicate BED files per RBP). Binding sites were further filtered by their log2 fold change (FC) to obtain $\sim$ 6,000 to 10,000 binding regions for each replicate. We next removed sites with length $>$ 0.75 percentile length and selected for each RBP the replicate set that contained the most regions, centered the sites, and extended them to make all sites of equal length. We chose a binding site length of 81 nt (40 nt extension up- and downstream of center position) and selected 30 RBP sets (some based on previous knowledge about binding preferences for comparison, the remaining ones random). To generate the eCLIP negative sets, RNAProt randomly selected sites based on two criteria: 1) their location on genes covered by eCLIP peak regions and 2) no overlap with

any eCLIP peak regions from the experiment. The same number of random negative and positive instances was used throughout the benchmarks.

Cross validation comparison

All three methods (GraphProt, DeepCLIP, RNAProt) were run using default parameters. For DeepCLIP we set patience (early stopping) to 20 and the maximum number of epochs to 200, since this setting was used the most in the DeepCLIP paper. For RNAProt we used a patience of 30 and maximum number of epochs to 200, which also is the tool default. An example call for DeepCLIP thus looked like this:

```
./DeepCLIP.py --runmode cv -n runtime_test_model
-P runtime_test_model_pred_fct
--predict_PFM_file pfms.json --sequences positives.5000.fa
--background_sequences negatives.5000.fa --num_epochs 200
--early_stopping 20 > runtime_test_model.log.txt
```

Likewise, an RNAProt call looks the following, with the dataset generated by `rnaprot gt` stored in `data_gt_out` used as input for training:

```
rnaprot train --in data_gt_out --out data_cv_train_out
--verbose-train --cv --only-seq
```

Hold-out comparison

For the hold-out comparison, DeepRAM was executed with its highest performing setting (ECBLSTM). This was achieved by calling DeepRAM with the following parameters (replace "data" with specific dataset ID):

```
python deepRAM.py --train_data data.train.gz --test_data data.test.gz
--data_type RNA --train True --evaluate_performance True
--model_path data.model.pkl --out_file data.predictions.txt --Conv True
--conv_layers 1 --Embedding True --RNN True --RNN_type BiLSTM --kmer_len 3
--stride 1 --word2vec_train True
--word2vec_model data.word2vec_train.model
```

We used 90% of a dataset for training, and the remaining 10% for testing. The same split was used for DeepRAM and RNAProt. For RNAProt, we used its option `--test-ids` to provide the same test IDs as used for DeepRAM for model training:

```
rnaprot train --in data_gt_out --out data_train_out --only-seq
--verbose-train --test-ids hold_out/data.test_ids
--val-size 0.2 --patience 50
```

Roquin CDE dataset preparation and prediction

To further assess the impact of adding structure information on RNAProt's predictive performance, we downloaded a dataset consisting of genomic regions containing potential human CDEs (constitutive decay elements) identified by [1] (Supplementary Table 6, table

"all"). A CDE consists of a short single hairpin with a tri-nucleotide loop that is preferably bound by the RBP Roquin. We then filtered the CDE containing sites by a minimum folding probability of 0.15, centered and extended them to 81 nt, and ran `rnaprot gt` with RNAplfold settings `--plfold-l 50`, `--plfold-w 70`, and `--plfold-u 3` to focus more on local hairpin structures. Finally we calculated the average model AUC with 10-fold cross validation, for both the sequence-only set and the sequence set with added structure information. We chose the most basic GRU model architecture (non-bidirectional GRU with one GRU layer, RNAProt default setting), corresponding to the following set parameters for training (`rnaprot train`):

```
--str-mode 1 --patience 30 --epochs 300 --batch-size 50
--lr 0.001 --weight-decay 0.0005 --n-rnn-layers 1 --n-hidden-dim 32
--dr 0.5 --model-type 1 --n-fc-layers 1
```

Note that we increased the maximum number of epochs from 200 to 300, which can help with smaller datasets like the described CDE set. We also ran GraphProt on the same set using the Galaxy version, to train a sequence and a structure model. For GraphProt we used the Galaxy default parameters.

For the window prediction, we used the UCP3 gene transcript (ENST00000314032.9). We trained a sequence model and a structure model, after excluding the CDE site on the UCP3 gene from the training set. For reporting peak regions, we used threshold levels `--thr 2` for the sequence and `--thr 1` for the structure model.

Runtime comparison

For the runtime comparison we took 5,000 positive and 5,000 negative training sequences, all with a length of 81 nt. Each tool was run three times using only the sequence information in train mode with default parameters. For GraphProt (sequence model mode), these are R: 1, D: 4, bitsize: 14, epochs: 10, and lambda: 0.001. For DeepCLIP and RNAProt, we used the default parameters together with a patience (early stopping) of 20 and a maximum number of 200 training epochs. For DeepCLIP, an example single model training call looked like this:

```
./DeepCLIP.py --runmode train -n TEST_MODEL
-P TEST_MODEL_PREDICTION_FUNCTION
--sequences positives.5000.fa --background_sequences negatives.5000.fa
--num_epochs 200 --early_stopping 20
```

References

[1] Johannes Braun, Sandra Fischer, Zhenjiang Z Xu, Hongying Sun, Dalia H Ghoneim, Anna T Gimbel, Uwe Plessmann, Henning Urlaub, David H Mathews, and Julia E Weigand. Identification of new high affinity targets for roquin based on structural conservation. *Nucleic acids research*, 46(22):12109–12125, 2018.

[2] Alexander Gulliver Bjørnholt Grønning, Thomas Koed Doktor, Simon Jonas Larsen, Ulrika Simone Spangsberg Petersen, Lise Lolle Holm, Gitte Hoffmann Bruun, Michael Birkerod Hansen, Anne-Mette Hartung, Jan Baumbach, and Brage Storstein Andresen. Deepclip: predicting the effect of mutations on protein–rna binding with deep learning. *Nucleic acids research*, 48(13):7099–7118, 2020.

[3] Daniel Maticzka, Sita J Lange, Fabrizio Costa, and Rolf Backofen. Graphprot: modeling binding preferences of rna-binding proteins. *Genome biology*, 15(1):R17, 2014.

[4] Cricket A Sloan, Esther T Chan, Jean M Davidson, Venkat S Malladi, J Seth Strattan, Benjamin C Hitz, Idan Gabdank, Aditi K Narayanan, Marcus Ho, Brian T Lee, et al. Encode data at the encode portal. *Nucleic acids research*, 44(D1):D726–D732, 2015.

[5] Ameni Trabelsi, Mohamed Chaabane, and Asa Ben-Hur. Comprehensive evaluation of deep learning architectures for prediction of dna/rna sequence binding specificities. *Bioinformatics*, 35(14):i269–i277, 2019.
