# Supplementary material for: RNAProt: an efficient and feature-rich RNA binding protein binding site predictor
Source: Gigascience. 2021 Aug 18;10(8):giab054. doi: 10.1093/gigascience/giab054 (PMC8372218; doi:10.1093/gigascience/giab054)
Supplement: giab054_Response_to_Reviewer_Comments_Original_Submission [file giab054_response_to_reviewer_comments_original_submission.pdf]

# Author's Response To Reviewer Comments

Close

Reviewer Comments and Replies
==============================

We would like to thank the reviewers for taking the time to review the manuscript and their constructive comments. We subsequently ran additional benchmarks and revised the manuscript (changes are marked in red). In the following, we are hoping to answer any outstanding questions.

1. Reviewer 1
=============

1.1 Major comments

About the negative sets: The author described the way of generating negative sets as "In case of sequences, negative sequences can be supplied or generated by k-nucleotide shuffling of positive sequences. In case of regions (BED format), negatives can be supplied or selected randomly from gene or transcript regions containing positive sites." Is there any literature or experimental evident to support this approach?

The idea was to offer the user several ways of generating a negative dataset. The common way to do this (e.g. in the GraphProt paper and other publications) is to select negative sites from gene regions which also include positive sites. This is also what we recommend: supply genomic or transcript regions and let RNAProt select the negatives using this strategy. This way we have evidence that the region is transcribed, and we can select negative sites within this region not overlapping with the RBP binding sites. If we would select sites randomly without the gene region restriction, we are less sure about the site being negative or not, since we do not have the expression evidence.

Alternatively, the user can supply his own negatives (e.g. for benchmarking existing datasets), or generate negatives from k-nucleotide shuffling of positive sequences (if FASTA sequences supplied). We have not evaluated whether this is better than sampling sites from the genome, since we only work with genomic or transcript regions. But it is likely not the optimal way to do this, since e.g. the commonly used di-nucleotide shuffling still produces sites that are somehow similar to the positive sites in content (i.e., identical regarding di-nt content).

As to whether this shuffling has been applied in literature, it is for example mentioned in the DeepRAM paper, quote:"The negative examples were generated by shuffling the positive sequences while matching dinucleotide composition." So using the k-nt shuffling option of RNAProt setting k=2 should therefore mimic the DeepRAM dataset generation. But as described, we recommend the random sampling approach. We therefore rewrote the section to make this more clear:

"In case genomic or transcript regions (BED format) are given as input, negatives can be supplied or selected randomly from gene or transcript regions containing positive sites (i.e., RBP binding sites identified by CLIP-seq). In

general, we recommend to supply BED regions, as this allows RNAProt to automatically generate a negative set by randomly sampling sites from the genome or transcriptome. By default, negative sites are sampled based on two criteria: 1) sampling only from gene regions covered by positive sites 2) no overlap with any positive site."

About the negative sets: "To generate the negative sets, we used RNAProt to randomly select sites based on two criteria: 1) their location on genes covered by positive regions and 2) no overlap with any positive regions (prefiltering) from the experiment." It is not clear that how to generate the negative sets. It seems contradictory between 1) covered and 2) no overlap.

Yes, the term "positive region" can easily be confused with "gene region containing positives". What we meant with "positive regions" was "positive sites", i.e., the binding sites identified by CLIP-seq. 1) means the location of the negative site should be on genes containing positive sites. 2) means that a negative sites should have no overlap with any positive site.

To make the description more clear, we changed the section to:

"To generate the negative sets, we used RNAProt which can automatically generate a set of random negative sites for a given set of positive input sites (i.e., RBP binding sites identified by CLIP-seq). By default, RNAProt randomly selects negative sites based on two criteria: 1) negative sites are sampled from gene regions containing positive sites and 2) a negative site should not overlap with any positive site. This setting was used to create the benchmark sets."

The training set and testing set are from the same RBPs. The deep learning model is trained on a specific protein and tested on the same protein. Without removing redundancy between the training set and the testing set, it is hard to avoid the overfitting problem due to on the same protein. The deep learning model only works in a specific protein, which is useless for a novel RBP.

This is the usual way the tools we refer and compare to work. Training a single model (on single RBP CLIP-seq data), and then use this model to predict new binding sites of the RBP, e.g. on gene regions or transcripts which are not present in the CLIP-seq experiment (== for which you have no CLIP-seq evidence). This is also often stated as the main motivation behind tools like RNAProt, DeepCLIP, etc.: since the CLIP-seq experiment can only recover sites for transcripts which are present in the experiment / the cell type / specific condition, a computational tool can help to predict missed out sites due to this expression dependency. Concerning multi-RBP / generic models, yes it would also be possible to combine datasets from several RBPs, and train one model on these, but this is not done so frequently.

In general, we try to avoid model overfitting during model training using standard regularization techniques, as also applied by the other deep learning methods. In addition, we compare our models using 10-fold cross validation, which is the standard way of estimating model generalization performance, and the widely acknowledged way it should be done (whenever possible). Here the trained model is evaluated on unseen test data for every fold. This way, potential overfitting can be uncovered (resulting in worse performance on the test set).

In figure 3, the lowest AUC of RNAProt is worse than DeepRAM.

The average AUC is almost the same as DeepRAM. It seems that we do not need another one. Could the author state the significant progress between RNAProt and DeepRAM?

We compared to DeepRAM based on the following reasons: it is easy to install and use, and it compares favorably to many other deep learning methods of the last years. However, deepRAM also has drawbacks: it does not offer 10-fold cross validation, and hyperparameter optimization cannot be disabled. This makes it difficult to do an objective comparison, since the other tools (GraphProt, DeepCLIP, RNAProt) use default (or fixed) hyperparameters in all the shown comparisons. One would expect that running a hyperparameter optimization for each model training would result in better performance than using default parameters, at least in some cases. So the comparison is not really objective (with a possible advantage introduced for DeepRAM), but as mentioned, the optimization cannot be disabled. The other problem with this is that the training runtime becomes very long. As mentioned in the paper: for a training set with 10,000 instances, DeepRAM took 5-6 hours, while RNAProt took 1-2 min. This is a striking advantage for RNAProt, allowing for on-the-fly-model training and testing. In addition, RNAProt supports various additional features like secondary structure or region features (even user-defined ones, which no other tool has), all of which DeepRAM does not offer, and as shown in the paper can further boost performance. Moreover, DeepRAM does not offer automatic negative set generation (apart from simple di-nt shuffling), leaving it to the user, who might not know how to do this. The combination of state-of-the-art performance (which we meant as: as good as or better than top competitors), ease-of-use, fast training and testing of various features is something that no other tool offers as far as we know. This basically also was the main motivation behind RNAProt: to offer the user a tool which is easy to use and well performing, but also feature-rich and runtime-efficient, things that we could not find combined in another tool. We therefore think that RNAProt is a significant progress. In addition, as we understood, GigaScience does not strictly focus on tools that out-perform all other tools, but also on other aspects which sets it apart from the compared tools. As stated in the Criteria section of GigaScience Technical Notes (https://academic.oup.com/gigascience/pages/technical_note):

"The tool or method described may be new, or an update or adaptation of an existing one. For software, this includes new functionality, improvements in performance or its interface, or a release on a new platform. The tool or method needs to have been tested, and properly compared to any existing tools or methods used by the community. It does not necessarily have to outperform existing approaches, but it should show innovation in the approach, implementation, or have added benefits that have been needed in this arena."

In this regard, RNAProt indeed has added benefits, which so far were not present in a single tool, or not at all (for the features only RNAProt offers). We made some changes to the text to emphasize the mentioned strengths more and make them more clear to the reader.

In the abstract:

"Since RNAProt supports various additional features (including user-defined ones which no other tool offers), we also present their influence on benchmark set performance. ... "

"RNAProt provides a complete framework for RBP binding site predictions, from dataset generation over model training to the evaluation of binding preferences and prediction. It offers state-of-the-art predictive performance as well as superior runtime efficiency, while at the same supporting more features and input types than any other tool available so far. RNAProt is easy to install and use, comes with a comprehensive documentation, and is accompanied by informative statistics and visualizations. All this makes RNAProt a valuable tool to apply in future RBP binding site research."

In the last paragraph of the introduction:

"The availability of a Conda package, which to our knowledge no other related deep learning tool offers, also allows for an easy integration into larger workflows, such as Snakemake pipelines [22] or inside the Galaxy framework [23]."

In the Supported features section:

"In addition, it also accepts user-defined region features (categorical or numerical, see documentation for details and examples), which no other tools so far offers."

"Note that genomic or transcript input sites are automatically extended on both sides (by window size) to get the most accurate structure predictions. This important feature is also not offered by any related tool."

Recently, two novel RNA-protein interaction binding site predictors PrismNet (Cell Res 2021 Feb 23;1-22) and PRIME-3D2D (Communications Biology, 16 July 2020) are published. However, this manuscript did not include these works. They reported that some RBPs showed dramatic performance improvement by PrismNet (e.g., METAP2: PrismNet = 0.90 vs GraphPort = 0.65; AUH: PrismNet = 0.89 vs GraphPort = 0.63; DDX55: PrismNet = 0.88 vs GraphPort = 0.65). Could the author benchmark RNAProt with PrismNet and PRIME-3D2D?

Thank you very much for the tool hints, we were not aware of these two tools. In the following we describe our experiences and if possible obtained results.

PrismNet
========
Source: https://github.com/kuixu/PrismNet

We downloaded the tool from Github, and installed it as described. We then tried to generate some datasets from the benchmark data we used in the manuscript to get AUC performances. In this process we ran into a number of issues:

- The installation as described did not work for us. We had to remove pytorch from the requirements.txt file, as it was already installed as advised. Also it would be nice to give a few more details on how to install pytorch with CUDA support (for less experienced users).

- There were errors in the dataset generation script (generate dataset.py), e.g. unset variables. A few days later some of them got fixed, so we tried again, but still we got the following error:

```
Traceback (most recent call last):
File ‘‘generate_dataset.py", line 52, in
structs = structs[i].split(’,’)
IndexError: list index out of range
```

We fixed this to create datasets in the required h5 format. But note that less experienced users might have problems with this and give up at this point already.

- It is necessary to provide in vivo structure data in order for PrismNet to work. However, there is no description anywhere on how to obtain it. Also,

this data might not be available for the datasets at hand. It would thus be better to make this input optional, which should be possible without much effort. For the comparison we set all in vivo structure data values to 0 (effectively disabling the feature), in order to use PrismNet with the hold out datasets from our manuscript (which we used to compare with deepRAM). Notably, PrismNet does not include a 10-fold cross validation mode, so we focussed on the sequence-only hold-out comparison.

- Concerning measuring the AUC performance, here we think PrismNet is making a quite problematic mistake. We checked the code and also tried some datasets, coming to the conclusion that PrismNet uses the provided test set as validation set and test set. This naturally results in higher performance measurements, as the tool optimizes the network parameters using the test set, and then estimates the generalization performance again on the test set. The canonical way to do this is to use a validation set independent of the test set in each fold. We don’t know how strongly this affected the PrismNet paper results, but in any case this is not the correct way to measure model generalization performance.

For the hold-out comparison, we therefore wrote a new dataset generation script, which takes care of the training, validation, and test splits. When benchmarking PrismNet on the hold-out sets just like for RNAProt and DeepRAM (using the same test sets), PrismNet performance is still very good, but on average a bit lower than the other two tools: on the first benchmark set (23 datasets) we get on average 86.46% for PrismNet, 87.42% for DeepRAM, and 87.50% for RNAProt. For the second set: 88.66% for PrismNet, 89.28% for DeepRAM, and 89.34% for RNAProt. See full results (Table 1 and Table 2) here: https://github.com/BackofenLab/RNAProt/blob/main/supplements/hold_out_comparison_prismnet.pdf

Due to the mentioned issues, the necessary manual workarounds to make the performance measurement work correctly, and the not significantly better performance we decided to not include or discuss the results in the manuscript.

PRIME-3D2D
==========
Webserver: www.rnabinding.com/PRIME-3D2D/PRIME-3D2D_genome.html

We checked the tool, and it seems there is only a webserver available, i.e. no manual installation possible (Webserver Link). This means we cannot run large benchmark comparisons (i.e., 10-fold cross validation or hold out with many datasets) as it would require a lot of manual file uploading, no parallelization etc., in general too time-consuming. Also, when using the webserver, we ran into several issues:

- It is not explained how to get the secondary structure information, which is required as input for the RNA part. The paper states that SeqFold was used for calculating the secondary structures. However, SeqFold seems to require experimental structure data, which is usually not available for a given RNA input sequence. And if it is available, it requires a considerable amount of preprocessing and knowledge how to do this. Therefore there should be a description on how to get this data and annotate the RNA, but this is missing. Alternatives are also not mentioned, e.g. it would be nice to get alternative recommendations like "for RNAs without experimental data, you can use tool ... ".

- The term "yeast genome" for running predictions on longer RNA sequences is confusing, as the user could think that only yeast data can be used. However (as we understood) any RNA sequences can be used (together with the pre-calculated RNA secondary structure information).

- When trying the example RNA on the website (YBL001C), and a PDB file from the PDB database (4ywq.pdb), we get the following uninformative error:

terminate called after throwing an instance of 'std::out_of_range'
what(): basic_string::at

It could be that we did something wrong here, but there is no complete or working input example given (PDB + structure) which could inform the user about possible mistakes. Also the meaning of "chain ID" and where to get this mandatory input is not mentioned anywhere. Even after looking up the term in the internet it was not clear to us. It might be easy for experts on PDB database / datasets, but for all others (we assume the average user) there should be at least a description on the site.

- When looking for a PDB file for the protein RC3H1 (Roquin 1) which we used in the manuscript for predictions, we found five different structures in the PDB database (3X1O, 4QIK, 4QIL, 4ULW, 4YWQ), all from the same domain but with varying lengths. It is not clear which ones to use, or whether it is advised to use only domains of RBPs, or instead only full-length RBP 3D structures. Again, there is no help / information regarding what to look for in the PDB databases. For users who are not familiar with this data it is quite difficult to know what to do here without any hints given.

We ran the webserver (yeast genome) with one of the five available RC3H1 PDB files, and the secondary structure predicted by RNAfold for the transcript ENST00000314032.9, which finished after ~ one day (we cannot tell exactly since there is no runtime information supplied). Regarding the interpretation of results, we ran into more issues:

Job Link:
http://www.rnabinding.com/PRIME-3D2D/upload/JkXQO7CsPO/

- The top 3 results are listed, but two of the text files do not contain any predicted binding sites (no "+" information given, which according to the HELP page mark predicted binding sites). Only the first one (Nrd1AYGL245W) contains this information.

- When searching for the predicted binding sites in the PRIME out file (storing all 22,337 predicted results), we can find predictions for the three sites listed there. However, Nrd1AYGL245W and SBP1AYGL245W each appear two times in the results file, with differing prediction results. It is not clear at all which predictions to use here. There is also no help or information given on the HELP page for this.

- Looking at the locations of the predicted binding sites, only one prediction out of the 5 predictions of the top 3 reported covers one of the two verified binding sites of the protein Roquin with "+" regions (SBP1AYGL245W "+" regions cover hairpin at 1,371 to 1,373 nt). On the other hand, there are more than 10 "+" regions in this case across the 2,277 nt long transcript sequence. In conclusion, the top 3 results (5 predictions) do not clearly support any of the two binding sites. By comparison, RNAProt with added structure information successfully identified the two verified binding sites, and predicted two more sites on the transcript.

Our idea was to compare the predicted Roquin binding sites of PRIME-3D2D with the ones reported for RNAProt in the manuscript. Unfortunately, there are many things unclear when using the webserver in its current state (as depicted). We think that a more detailed HELP page and a clearer output format would help the user a lot. The method for sure is interesting, but unfortunately due to the observed problems this is all we were able to do as far as comparing PRIME-3D2D with RNAProt.

1.2 Minor comments

What is exon-intron regions feature? How to calculate it? Why this feature works?

The description of this feature and its calculation can be found in the Methods section "Exon-intron annotation". Since we are usually dealing with genomic regions / binding sites, these can be annotated on a position-wise basis, by overlapping them with exon and intron regions, which are stored in GTF annotation files, similarly to the other region annotations. Many RBPs have tendencies to bind to exonic or intronic regions. For example, there are RBPs which are involved in post-transcriptional gene regulation which therefore often bind to exonic regions (the spliced RNA basically). Such region annotation information can be utilized in model training.

What is transcript regions feature? How to calculate it? Why this feature works?

The description of this feature and its calculation can be found in the Methods section "Transcript region annotation". The annotation works the same way as with the exon-intron region features. A GTF genomic annotations file usually contains information about transcript CDS or UTR (5’ or 3’) regions, so by overlapping the RBP binding sites with these regions, we can make a position-wise annotation of these regions. For example, certain RBPs tend to bind to 3’UTR regions. It can thus make sense to combine this information with the basic sequence information. This information has also been used in the publication for the tool DeepRiPe. Unfortunately, the tool is not usable in its current state, as there is no manual, installation description, or main executable file (see Github repo). This again exemplifies what got us motivated to implement RNAProt.

DeepRiPe paper:
https://genome.cshlp.org/content/30/2/214
DeepRiPe Github Repo:
https://github.com/ohlerlab/DeepRiPe

2. Reviewer 2
=============

2.1 Major comments

First, as mentioned by the authors, there are already many methods published for RBP binding site prediction. The contribution and novelty of RNAProt may need to be clearly described and discussed in details.

While various methods have been published over the years, many problems arise upon closer inspection. We tried to point out these problems shortly in the abstract, and in the last two sections of the introduction, and how RNAProt helps with these. The main problems we encountered were:

- Problems / inability of installation and / or no usage instructions. This is true not only for older but also more recently published tools. E.g.

DeepRiPe, where there is no manual, help page, or installation description (see Github repo) Or similarly RPI-Net (RNAonGraph), see the following links:

DeepRiPe:
https://genome.cshlp.org/content/30/2/214
https://github.com/ohlerlab/DeepRiPe

RPI-Net (RNAonGraph):
https://doi.org/10.1093/bioinformatics/btaa456
https://github.com/HarveyYan/RNAonGraph

While such papers undoubtedly offer valuable new insights, tools with missing installation or usage instructions cannot really be classified as tools in a sense that a user can readily use them, where clear installation or usage instructions become mandatory. Consequently, we can also not compare to these tools. RNAProt on the other hand is easy to install, as a conda package also exists, which to our knowledge no other recent deep learning method offers. A Conda package also allows for easy / automatic tool integration into bigger workflows, e.g. into Snakemake pipelines, or into the Galaxy framework. Offering webservers can mitigate this (to make the tool more accessible for users with less experience), like DeepCLIP does. But here we also encountered problems. For example, we tried to train a DeepCLIP model (with a relatively small training dataset) using the DeepCLIP webserver, but this job did not finish even after several weeks.

- Efficiency problems regarding runtime (see runtime comparison with the recent deep learning tools DeepRAM and DeepCLIP). For example, training a single model took 5-6 hours for DeepRAM (already after changing the code to half of the hyperparameter optimization iterations) vs 1-2 min for RNAProt. DeepCLIP has a shorter training time, but 35 min vs. 1.5 minutes is still a huge difference (even more so when running 10-fold cross validation, resulting in a runtime for DeepCLIP of close to 6 h, compared to 10-15 min for RNAProt).

- Limited input type and feature support. This includes features for learning, but also tool settings for customization (see Online Documentation). While there have been certain specialized tools published in the past (e.g. using conservation information or secondary structure information), there is to our knowledge no tool which offers various combinations of them.

In addition, RNAProt supports user-defined region features (i.e., any genomic or transcript region annotation), which no other tool so far offers. This would make it possible to instantly add e.g. in vivo structure data, without modifying the code (since numerical position-wise values for annotation are also supported). Concerning input types, RNAProt supports sequences, genomic regions, and also transcript regions. The third one no other tool is offering, while the second one is only supported by a few (DeepCLIP is the only one we know of, but e.g. DeepRAM does not). The same holds for automatic negative set generation, which most of the tools do not offer (DeepCLIP for example does, but there you do not have much control over the process). In RNAProt, you can e.g. also specify which genomic regions to exclude (e.g. if you have CLIP data for the same RBP from other experiments), extract additional negatives, or filter out low complexity negative sites.

All this in our opinion are valuable benefits, with the goal to give the user an easy-to-use tool with strong performance and improved runtime efficiency for fast model training and prediction. The ease-of-use is especially important for less experienced users, who might not know e.g. how to best generate a negative set on their own. We therefore also added a comprehensive examples section and a Documentation to the Github site. We think this approach is also

in agreement with the publishing policy of GigaScience (e.g. the "added benefits" criterion), as stated in the Criteria section of GigaScience Technical Notes (https://academic.oup.com/gigascience/pages/technical_note):

"The tool or method described may be new, or an update or adaptation of an existing one. For software, this includes new functionality, improvements in performance or its interface, or a release on a new platform. The tool or method needs to have been tested, and properly compared to any existing tools or methods used by the community. It does not necessarily have to outperform existing approaches, but it should show innovation in the approach, implementation, or have added benefits that have been needed in this arena."

To enforce the contribution of RNAProt, we rewrote the abstract to:

"Since RNAProt supports various additional features (including user-defined ones which no other tool offers), we also present their influence on benchmark set performance. ... "

"RNAProt provides a complete framework for RBP binding site predictions, from dataset generation over model training to the evaluation of binding preferences and prediction. It offers state-of-the-art predictive performance as well as superior runtime efficiency, while at the same supporting more features and input types than any other tool available so far. RNAProt is easy to install and use, comes with a comprehensive documentation, and is accompanied by informative statistics and visualizations. All this makes RNAProt a valuable tool to apply in future RBP binding site research."

And in the last paragraph of the introduction:

"The availability of a Conda package, which to our knowledge no other related deep learning tool offers, also allows for an easy integration into larger workflows, such as Snakemake pipelines [22] or inside the Galaxy framework [23]."

"Note that genomic or transcript input sites are automatically extended on both sides (by window size) to get the most accurate structure predictions. This important feature is also not offered by any related tool."

As for the discussion / comparison to other tools, we discussed possible benefits and drawbacks of more complex architectures such as the one used by deepRAM in the "Hold-out validation comparison" results section, hoping that these are sufficient to understand the benefits and possible drawbacks.

Second, one main conclusion from the deepRAM study is that hybrid CNN/RNN architectures outperformed other methods with sufficient training data. DeepCLIP also used a combination of CNN and BLSTM layers to predict the effects of mutations on protein-RNA binding. I wonder if the performance of RNAProt could be further enhanced by including an additional layer or layers of CNN.

This would be interesting to try yes, but as we have shown in the benchmarks, the added CNN layer does not seem help much in boosting the predictive performance of DeepCLIP / DeepRAM in comparison to RNAProt, at least not with the datasets we tried (53 CLIP-seq datasets produced by various protocols: eCLIP, PARCLIP, iCLIP, HITS-CLIP). Since we used only sequence information for all tools in the comparison, the presented information is exactly the same for all tools in the comparison. We therefore can assume that any potential benefits of the added CNN (if present) should become noticeable in the results.

As discussed in the paper ("Hold-out validation comparison" results section), the effect might be more drastic when including bigger datasets. But CLIP-Seq experiments usually do not yield > 10,000 binding sites for a certain RBP, and it is also questionable how many of these are true binding sites. As described, e.g. the standard filtering applied by the ENCODE consortium (hosting the eCLIP datasets) usually results in (much) less sites for analysis.

RNAProt does support hyperparameter optimization (HPO) using BOHB, which also includes the addition of more layers (e.g. two RNN layers, type of RNN (GRU, LSTM), bidirectional information, an additional layer after the RNN layers, or an embedding layer before the RNN layers). When testing the HPO (for ~ 10 datasets), we noticed that the AUC performance can increase up to ~ 3-4 %, but most of the time it is less than the variation one gets from 10-fold cross validation, thus not significantly different from the default parameters. So for the RNN part we can assume that adding more layers usually does not change the performance much. This also tells us that the chosen architecture and hyperparameter settings are a good choice for CLIP-seq data. As for an added CNN, this could of course help depending on the type of data / dataset, but then the runtime will also increase again, making the tool less usable for on-the-fly model training and hypothesis testing. Consequently, the tool would also become less distinguishable from the other tools. We therefore decided to use a solely RNN-based architecture, which showed to offer both fast runtime and competitive performance.

3. Reviewer 3
=============

3.1 Major comments

I see the same authors also present a graph neural network-based method GraphProt2 to predict RBP binding sites, although the method is still a preprint and not published. However, I did not see the authors compare their proposed method RNAProt with GraphProt2 and its baseline method iDeepS, whose architecture is also used by DeepCLIP.

Yes, we decided to go for an RNN-based architecture instead of the GNN architecture which was used for GraphProt2. While the GNN architecture in principle has great potential, namely the natural encoding of base pair information into graphs which other architectures cannot do, including the base pair information did not boost the performance the way we hoped for. As discussed in the GraphProt2 preprint, there could be many reasons for this, e.g. a suboptimal encoding, CLIP protocol biases, or the computational prediction of base pairs. Moreover, the runtime was not as good as RNAProt with its solely RNN-based architecture, which led us to focus on RNAProt instead. We still want to investigate which structure encoding works better in the case of GNNs, e.g. predicted vs in vivo structure data, but this will take more time. Also, as GraphProt2 is not published in a peer-reviewed journal, we assume it is unusual (and possibly also not accepted by the journal) to compare it with RNAProt.

As for iDeepS, both DeepCLIP and deepRAM showed that they compare favorably to iDeepS in their own publications, among many other tools. We therefore decided to choose DeepCLIP and deepRAM for comparison, since these are also more recent tools, using similar architectures.

RNAProt uses RNNs for making classification, how about CNNs or

CNN+RNNs, the authors need show that the selected RNN is superior to others.

Based on the benchmark results, we can conclude that the RNN-based architecture of RNAProt performs more or less equally as good or better than the CNN+RNN architectures used by DeepCLIP and deepRAM. In other words, the added CNN layer does not seem help much in boosting the predictive performance of DeepCLIP / DeepRAM in comparison to RNAProt, at least not with the datasets we tried (53 CLIP-seq datasets produced by various protocols: eCLIP, PARCLIP, iCLIP, HITS-CLIP). Although it is hard to tell how strong the contribution of the network architecture is, it should be noticeable if there is a significant benefit, since we only use the sequence information for each tool in the comparison (so all tools see exactly the same information, without any variation coming from additional features).

As discussed in the paper (see "Hold-out validation comparison" results section), the effect of more complex networks might be more drastic when including bigger datasets. But CLIP-Seq experiments usually do not yield > 10,000 binding sites for a certain RBP, and it is also questionable how many of these are true binding sites. As described, e.g. the standard filtering applied by the ENCODE consortium (hosting the eCLIP datasets) usually results in (much) less sites for analysis.

As for the selected RNN-based architecture: RNAProt does support hyperparameter optimization (HPO) using BOHB, which also includes the addition of more layers (e.g. two RNN layers, an additional layer after the RNN layers, or an embedding layer before the RNN layers), or to change the type of RNN (LSTM instead of GRU), or bidirectional information. When testing the HPO (for ~ 10 datasets), we noticed that the AUC performance can increase up to ~ 3-4 %, but most of the time (i.e., for most datasets) it is less than the variation one gets from 10-fold cross validation, thus not significantly different from the default parameters. So for the RNN part we can assume that adding more layers usually does not significantly change the performance. This also tells us that the chosen architecture and hyperparameter settings are a good choice for CLIP-seq data. Less layers of course also help with the runtime, so they are very welcome as long as predictive performance does not suffer. Another hint that solely RNN-based architectures are a good choice for CLIP-seq data comes from the DeepRAM paper, where KEGRU (solely RNN-based) is the third best tool on CLIP-seq data (Figure 2c, average AUC performance, with the DeepRAM architecture we compare to at first place), even though it was implemented for ChIP-seq data and thus not optimized for CLIP-seq.

DeepRAM paper:
https://doi.org/10.1093/bioinformatics/btz339

As for an added CNN, this could of course help depending on the type of data / dataset, but then the runtime will also increase again, making the tool less usable for on-the-fly model training and hypothesis testing. Consequently, the tool would also become less distinguishable from the other tools. We therefore decided to use an solely RNN-based architecture, which showed to offer both fast runtime and competitive performance. These were also part of our main goals: to implement a runtime-efficient tool with competitive performance. RNAProt manages to be that, plus it offers additional features (more than other tools plus some no other tool offers), more input types (e.g. transcript region input no other tool offers), automatic negative set generation with options (most tools do not offer this), and easy installation (a Conda package is available for easy integration into bigger workflows, which to our knowledge other related deep learning tools also do not offer). We therefore think that RNAProt is a significant progress. We made some changes to the text to emphasize the mentioned strengths more and make them more clear to the reader.

In the abstract:

"Since RNAProt supports various additional features (including user-defined ones which no other tool offers), we also present their influence on benchmark set performance. ... "

"RNAProt provides a complete framework for RBP binding site predictions, from dataset generation over model training to the evaluation of binding preferences and prediction. It offers state-of-the-art predictive performance as well as superior runtime efficiency, while at the same supporting more features and input types than any other tool available so far. RNAProt is easy to install and use, comes with a comprehensive documentation, and is accompanied by informative statistics and visualizations. All this makes RNAProt a valuable tool to apply in future RBP binding site research."

In the last paragraph of the introduction:

"The availability of a Conda package, which to our knowledge no other related deep learning tool offers, also allows for an easy integration into larger workflows, such as Snakemake pipelines [22] or inside the Galaxy framework [23]."

In the Supported features section:

"In addition, it also accepts user-defined region features (categorical or numerical, see documen-tation for details and examples), which no other tools so far offers."

"Note that genomic or transcript input sites are automatically extended on both sides (by window size) to get the most accurate structure predictions. This important feature is also not offered by any related tool."

Regarding superior performance: as we understood, GigaScience does not strictly focus on tools that out-perform all other tools, but also on other aspects which sets it apart from the compared tools. As stated in the Criteria section of GigaScience Technical Notes (https://academic.oup.com/gigascience/pages/technical_note):

"The tool or method described may be new, or an update or adaptation of an existing one. For software, this includes new functionality, improvements in performance or its interface, or a release on a new platform. The tool or method needs to have been tested, and properly compared to any existing tools or methods used by the community. It does not necessarily have to outperform existing approaches, but it should show innovation in the approach, implementation, or have added benefits that have been needed in this arena."

In this regard, we think that RNAProt indeed has added benefits (besides performance), which so far were not present in a single tool, or for the features only RNAProt offers not at all.

How does RNAProt handle sequences with variable lengths, and how are additional features extracted for them?

Since RNAProt utilizes a solely RNN-based architecture, it can handle input sequences of variable length without any further preprocessing required (which must be done for CNN methods like DeepCLIP and deepRAM since these only allow fixed-length inputs). This was also one reason why we decided to use an RNN type (besides faster runtime and the shown competitive performance). Additional features are extracted as described in the Methods section "Supported features". The extraction process differs depending on the feature (see

Table 1 for supported features). For example, there are region features like transcript regions, exon-intron regions, or user-defined regions which the user can supply (any number of them). These are overlapped with the input sites (BED format), using the information in the provided GTF file. So if a position in a given genomic (or transcript) input site overlaps with a certain region annotation (for example an exon), this position gets an exon label assigned (here we use one-hot encoding, but can also be numerical for user-defined region features). Position-wise repeat region information is extracted directly from the supplied .2bit genomic sequence file. Conservation scores on the other hand are extracted from bigWig files containing this conservation information. As for the structure feature, position-wise unpaired probabilities are computed using RNAplfold. For this to be precise, RNAProt extends the input site by the window length of RNAplfold on both sides with its genomic (or transcript) context, to calculate the probabilities for each site position. This is also an important feature no other tool offers as far as we know.

To elaborate the variable lengths feature a bit more, we expanded the training set generation section part to:

"The number of generated negative sites can be further specified, as well as regions from which not to extract them. Output site lengths can be of variable or fixed size, and various filtering options are available to filter the sites by score, sequence complexity, region, or length. Concerning site lengths, RNAProt can train and predict on sequences of variable length due to its solely RNN-based architecture. For CNN-based methods this is usually not the case (unless the method internally applies padding before training and predicting). To keep datasets compatible with other tools, RNAProt therefore offers both variable and fixed-size outputs. Depending on the input type (see Table 1), different additional features can be selected for annotating the positive and negative sites (see Supported features section for more details)."

If a full-length sequence is given, how RNAProt can be applied to determine whether an RBP will interact it or not?

RNAProt offers two kinds of prediction modes (see rnaprot predict --h and also the run examples in the manual which demonstrate this). In the first mode, RNAProt scores the full-length input sequences or sites, so one score per site is obtained. In the second mode, RNAProt is using a sliding window approach, meaning that it scores each sliding window (step size 1), and only returns the peak regions within sliding windows that have scores higher than a set threshold. Peak region means that instead of reporting the full length window, RNAProt looks at the saliency profile, and extracts the region with the highest saliencies within the window. Currently there are 3 threshold settings (--thr), which correspond to relaxed, standard, and strict filtering. The thresholds for these settings are taken directly from the positive set on which the model was trained on, so they are not fixed but different for every model. This way we want to ensure to only extract sites which have high scores compared to the positive training set (since model scores are always relative and depend on the trained model). For example, in the standard setting, we use the 50th percentile score of the positive training set as a threshold. This means that the reported sites have scores that are as good or higher than the bottom 50 percent scores of the positive training set sites. In addition, the output also contains a p-value, which indicates how good the score precisely is compared to the whole positive set (low p-values = higher scores). As shown in the results section "Structure information can increase specificity", this window strategy successfully predicted the hairpin loop binding sites of the RBP Roquin.

In Figure 4, the authors demonstrate that RNAProt runs faster than

others, does the time contains additional features extraction and structure prediction time? For longer sequences, structure prediction is very time-consuming? Thus, what is the sequence length here for benchmarking the running time?

All sites in the benchmark sets were normalized to a length of 81 nt. For the runtime comparison (and also all the benchmark comparisons with the other tools), we use RNAProt without additional features (so no structure information, only sequence features). Regardless of whether to use additional features or not, a dataset has to be generated first (using rnaprot gt). In the case of sequences as input, this takes only a couple of seconds. But of course as soon as we supply BED regions as input and demand from RNAProt to generate a fitting negative set, this process takes longer (extraction of sites from the genome, filtering). We did not include this time in the benchmark, since for the other tools you would also have to do this preprocessing before training a model (i.e., creating a training set). As for the additional features: these are also calculated when running rnaprot gt, and not in the training step (rnaprot train). This way RNAProt needs to calculate the features of interest only once, and then for training the user can decide which feature or feature combinations to try (feature information is stored in the training set folder). Since RNAProt model training is fast (regardless of what features are set), this also allows to quickly test different features or combinations of them.

Calculating the structure information during rnaprot gt indeed takes some time, as RNAProt uses RNAplfold, whose runtime depends on the number of input sites, their length, and the settings such as window length, and maximum base pair span. Also note that RNAProt extends the input sites (if genomic or transcript) by the RNAplfold window length on both sides, to make the structure calculations more accurate (which again leads to longer runtime). For a dataset with 10,000 sites (5,000 positive input sites, 5,000 negatives generated by RNAProt during the run), and default RNAplfold settings (--plfold-u 3, --plfold-l 50, --plfold-w 70) rnaprot gt with structure feature calculation enabled takes about 5 min on the benchmark PC (Intel i7-8700k). Calculating the other features is faster, so for the same dataset and calculating all the standard features (structure, exon-intron, transcript, repeat regions, conservation information) takes about 10 min.

In Figure 5, RNAProt can extract motifs, how is the single nt mut logo derived? More details should be given. In addition, TOMTOM may be used to compare the extract motifs against known motifs.

Yes, this was only briefly and not clearly described in the Visualization Section. We rewrote the part of the section to:

"In contrast, in silico mutagenesis treats the network as a black box, where the input sequence is mutated at each position (three mutations possible at each position since there are three non-wildtype nucleotides), and the mutated sequences are scored by the network. For example, given a sequence AC, the mutated sequences would be CC, GC, UC, AA, AG, and AU. For a sequence of length n we thus need to generate 3*n mutated sequences to calculate scores for. The score difference (mutated sequence score minus wildtype sequence score) is then plotted for each mutated nucleotide at each position, with the height of the nucleotide corresponding to the score difference (see Figures 5 and 7 for examples). This difference can be positive (i.e., the mutation increases the whole-site score) or negative (i.e., the mutation decreases the whole-site score). This way, both visualizations help in understanding what parts the model regards as important in a given sequence."

Thank you very much for the hint with TOMTOM to compare found motifs. We designed the logo visualization to get a compact idea on global binding

preferences of the model (similar to the way GraphProt visualized binding preferences). To generate the logo, the region of highest importance (in our case most salient position + extension on both sides) is taken for a specified number of highest-scoring sites. This way, one logo is generated for each dataset (number of sites to extract from and logo size can be set in rnaprot eval). This means the logo is not a motif in the traditional sense, as it can also represent a mixture of several motifs (motif in sense of a sequence pattern with distinct nucleotide composition, identified by a motif finder). However, it should also be possible to extract standard motifs by taking the peak regions (high scoring sites) identified by RNAProt (using rnaprot predict, e.g. running window predictions on a sufficient number of transcripts or longer gene sequences), and input these to motif finders like DREME or similar tools. The comparison with known motifs through TOMTOM could then be done as part of a larger pipeline, as a post-processing step.

Close
