# Supplementary material for: RNAProt: an efficient and feature-rich RNA binding protein binding site predictor
Source: Gigascience. 2021 Aug 18;10(8):giab054. doi: 10.1093/gigascience/giab054 (PMC8372218; doi:10.1093/gigascience/giab054)
Supplement: giab054_Response_to_Reviewer_Comments_Revision_1 [file giab054_response_to_reviewer_comments_revision_1.pdf]

Author's Response To Reviewer Comments

Close

> To sum it up, your manuscript is acceptable for publication in GigaScience, in principle.

> I am happy to say that the editorial board members agree with your arguments, as the model's main use is to find unknown binding sites or bindable RNA sequences from *known* RBPs (rather than identifying new RBPs), and as such, the data splitting scheme and cross validation is adequate, in the opinion of our Editorial Board.

We want to thank the editors and reviewers for revising the manuscript!

> Before publication, our data curators may take a snapshot of the code (and other supporting data) for our repository GigaDB, they will contact you shortly. Please include the GigaDB dataset (including the doi link) in your bibliography and cite it from your "availability" section. (you will get the exact citation details and doi from our curators).

We modified the Availability section (changes marked in red) and added the needed GigaDB citation.

Close
