# Supplementary material for: RNAProt: an efficient and feature-rich RNA binding protein binding site predictor
Source: Gigascience. 2021 Aug 18;10(8):giab054. doi: 10.1093/gigascience/giab054 (PMC8372218; doi:10.1093/gigascience/giab054)
Supplement: giab054_Response_to_Reviewer_Comments_Revision_2 [file giab054_response_to_reviewer_comments_revision_2.pdf]

Author's Response To Reviewer Comments

Close

We added the Zenodo and GitHub citations, as well as the ORCID links on the title page.

Close
