# Supplementary material for: RNAProt: an efficient and feature-rich RNA binding protein binding site predictor
Source: Gigascience. 2021 Aug 18;10(8):giab054. doi: 10.1093/gigascience/giab054 (PMC8372218; doi:10.1093/gigascience/giab054)
Supplement: giab054_Reviewer_1_Report_Original_Submission — Shiyong Liu -- 4/26/2021 Reviewed [file giab054_reviewer_1_report_original_submission.pdf]

Reviewer Report

Title: RNAProt: An efficient and feature-rich RNA binding protein binding site predictor

Version: Original Submission **Date:** 4/26/2021

Reviewer name: Shiyong Liu

Reviewer Comments to Author:

The author presented an RNA binding protein binding site predictor RNAProt, which is based on RNNs. They compare RNAProt with one traditional machine learning approach and two deep learning methods and claim that its state-of-the-art predictive performance. It seems promising; however, I have the following comments.

Major comments:

1) Question 1 about the negative sets: The author described the way of generating negative sets as "In case of sequences, negative sequences can be supplied or generated by k-nucleotide shuffling of positive sequences. In case of regions (BED format), negatives can be supplied or selected randomly from gene or transcript regions containing positive sites." Is there any literature or experimental evident to support this approach?

2) Question 2 about the negative sets : "To generate the negative sets, we used RNAProt to randomly select sites based on two criteria: 1) their location on genes covered by positive regions and 2) no overlap with any positive regions (prefiltering) from the experiment." It is not clear that how to generate the negative sets. It seems contradictory between 1) covered and 2) no overlap.

3) The training set and testing set are from the same RBPs. The deep learning model is trained on a specific protein and tested on the same protein. Without removing redundancy between the training set and the testing set, it is hard to avoid the overfitting problem due to on the same protein. The deep learning model only works in a specific protein, which is useless for a novel RBP.

4) In figure 3, the lowest AUC of RNAProt is worse than DeepRAM. The average AUC is almost the same as DeepRAM. It seems that we do not need another one. Could the author state the significant progress between RNAProt and DeepRAM?

5) Recently, two novel RNA-protein interaction binding site predictors PrismNet (Cell Res 2021 Feb 23;1-22) and PRIME-3D2D (Communications Biology, 16 July 2020) are published. However, this manuscript did not include these works. They reported that some RBPs showed dramatic performance improvement by PrismNet (e.g., METAP2: PrismNet = 0.90 vs GraphPort = 0.65; AUH: PrismNet = 0.89 vs GraphPort = 0.63; DDX55: PrismNet = 0.88 vs GraphPort = 0.65). Could the author benchmark RNAProt with PrismNet and PRIME-3D2D?

Minor comments:

1) What is exon-intron regions feature? How to calculate it? Why this feature works?

2) What is transcript regions feature? How to calculate it? Why this feature works?

Level of Interest

Please indicate how interesting you found the manuscript: Choose an item.

Quality of Written English

Please indicate the quality of language in the manuscript: Choose an item.

Declaration of Competing Interests

Please complete a declaration of competing interests, considering the following questions:

- Have you in the past five years received reimbursements, fees, funding, or salary from an organisation that may in any way gain or lose financially from the publication of this manuscript, either now or in the future?
- Do you hold any stocks or shares in an organisation that may in any way gain or lose financially from the publication of this manuscript, either now or in the future?
- Do you hold or are you currently applying for any patents relating to the content of the manuscript?
- Have you received reimbursements, fees, funding, or salary from an organization that holds or has applied for patents relating to the content of the manuscript?
- Do you have any other financial competing interests?
- Do you have any non-financial competing interests in relation to this paper?

If you can answer no to all of the above, write 'I declare that I have no competing interests' below. If your reply is yes to any, please give details below.

I declare that I have no competing interests

I agree to the open peer review policy of the journal. I understand that my name will be included on my report to the authors and, if the manuscript is accepted for publication, my named report including any attachments I upload will be posted on the website along with the authors' responses. I agree for my report to be made available under an Open Access Creative Commons CC-BY license (http://creativecommons.org/licenses/by/4.0/). I understand that any comments which I do not wish to be included in my named report can be included as confidential comments to the editors, which will not be published.

Choose an item.

To further support our reviewers, we have joined with Publons, where you can gain additional credit to further highlight your hard work (see: https://publons.com/journal/530/gigascience). On publication of this paper, your review will be automatically added to Publons, you can then choose whether or not to claim your Publons credit. I understand this statement.

Yes Choose an item.
