# Supplementary material for: RNAProt: an efficient and feature-rich RNA binding protein binding site predictor
Source: Gigascience. 2021 Aug 18;10(8):giab054. doi: 10.1093/gigascience/giab054 (PMC8372218; doi:10.1093/gigascience/giab054)
Supplement: giab054_Reviewer_3_Report_Original_Submission — XIAOYONG Pan -- 5/1/2021 Reviewed [file giab054_reviewer_3_report_original_submission.pdf]

Reviewer Report

Title: RNAProt: An efficient and feature-rich RNA binding protein binding site predictor

Version: Original Submission **Date:** 5/1/2021

Reviewer name: XIAOYONG Pan

Reviewer Comments to Author:

The authors an efficient and feature-rich computational RBP binding site prediction framework RNAProt based on recurrent neural networks with the support of additional features, it starts from data generation to performance evaluation.

Major:

1. I see the same authors also present a graph neural network-based method GraphProt2 to predict RBP binding sites, although the method is still a preprint and not published. However, I did not see the authors compare their proposed method RNAProt with GraphProt2 and its baseline method iDeepS, whose architecture is also used by DeepCLIP.
2. RNAProt uses RNNs for making classification, how about CNNs or CNN+RNNs, the authors need show that the selected RNN is superior to others.
3. How does RNAProt handle sequences with variable lengths, and how are additional features extracted for them?
4. If a full-length sequence is given, how RNAProt can be applied to determine whether an RBP will interact it or not?
5. In Figure 4, the authors demonstrate that RNAProt runs faster than others, does the time contains additional features extraction and structure prediction time? For longer sequences, structure prediction is very time-consuming? Thus, what is the sequence length here for benchmarking the running time?
6. In Figure 5, RNAProt can extract motifs, how is the single_nt_mut logo derived? More details should be given. In addition, TOMTOM may be used to compare the extract motifs against known motifs.

Level of Interest

Please indicate how interesting you found the manuscript: Choose an item.

Quality of Written English

Please indicate the quality of language in the manuscript: Choose an item.

Declaration of Competing Interests

Please complete a declaration of competing interests, considering the following questions:

- Have you in the past five years received reimbursements, fees, funding, or salary from an organisation that may in any way gain or lose financially from the publication of this manuscript, either now or in the future?
- Do you hold any stocks or shares in an organisation that may in any way gain or lose financially from the publication of this manuscript, either now or in the future?
- Do you hold or are you currently applying for any patents relating to the content of the manuscript?
- Have you received reimbursements, fees, funding, or salary from an organization that holds or has applied for patents relating to the content of the manuscript?
- Do you have any other financial competing interests?
- Do you have any non-financial competing interests in relation to this paper?

If you can answer no to all of the above, write 'I declare that I have no competing interests' below. If your reply is yes to any, please give details below.

I declare that I have no competing interests

I agree to the open peer review policy of the journal. I understand that my name will be included on my report to the authors and, if the manuscript is accepted for publication, my named report including any attachments I upload will be posted on the website along with the authors' responses. I agree for my report to be made available under an Open Access Creative Commons CC-BY license (http://creativecommons.org/licenses/by/4.0/). I understand that any comments which I do not wish to be included in my named report can be included as confidential comments to the editors, which will not be published.

Choose an item.

To further support our reviewers, we have joined with Publons, where you can gain additional credit to further highlight your hard work (see: https://publons.com/journal/530/gigascience). On publication of this paper, your review will be automatically added to Publons, you can then choose whether or not to claim your Publons credit. I understand this statement.

Yes Choose an item.
